# Inducible Stem-Cell-Derived Embryos Capture Mouse Morphogenetic Events *In Vitro*

**DOI:** 10.1016/j.devcel.2020.12.004

**Published:** 2021-02-08

**Authors:** Gianluca Amadei, Kasey Y.C. Lau, Joachim De Jonghe, Carlos W. Gantner, Berna Sozen, Christopher Chan, Meng Zhu, Christos Kyprianou, Florian Hollfelder, Magdalena Zernicka-Goetz

**Affiliations:** 1 Department of Physiology, Development and Neuroscience, University of Cambridge, Cambridge CB2 3EG, UK; 2 Department of Biochemistry, University of Cambridge, Cambridge CB2 1GA, UK; 3 Division of Biology and Biological Engineering, Caltech, Pasadena, CA 91125, USA

**Keywords:** induced ETX-embryos, stem cells, gastrulation, EMT, embryogenesis, gastruloid

## Abstract

The development of mouse embryos can be partially recapitulated by combining embryonic stem cells (ESCs), trophoblast stem cells (TS), and extra-embryonic endoderm (XEN) stem cells to generate embryo-like structures called ETX embryos. Although ETX embryos transcriptionally capture the mouse gastrula, their ability to recapitulate complex morphogenic events such as gastrulation is limited, possibly due to the limited potential of XEN cells. To address this, we generated ESCs transiently expressing transcription factor Gata4, which drives the extra-embryonic endoderm fate, and combined them with ESCs and TS cells to generate induced ETX embryos (iETX embryos). We show that iETX embryos establish a robust anterior signaling center that migrates unilaterally to break embryo symmetry. Furthermore, iETX embryos gastrulate generating embryonic and extra-embryonic mesoderm and definitive endoderm. Our findings reveal that replacement of XEN cells with ESCs transiently expressing Gata4 endows iETX embryos with greater developmental potential, thus enabling the study of the establishment of anterior-posterior patterning and gastrulation in an *in vitro* system.

## Highlights

• Stem cells generate mouse-embryo-like structures with improved potential
• These structures undertake anterior visceral endoderm formation and gastrulation
• Single-cell sequencing shows improved resemblance to mouse embryo

## Graphical Abstract

**Figure:**
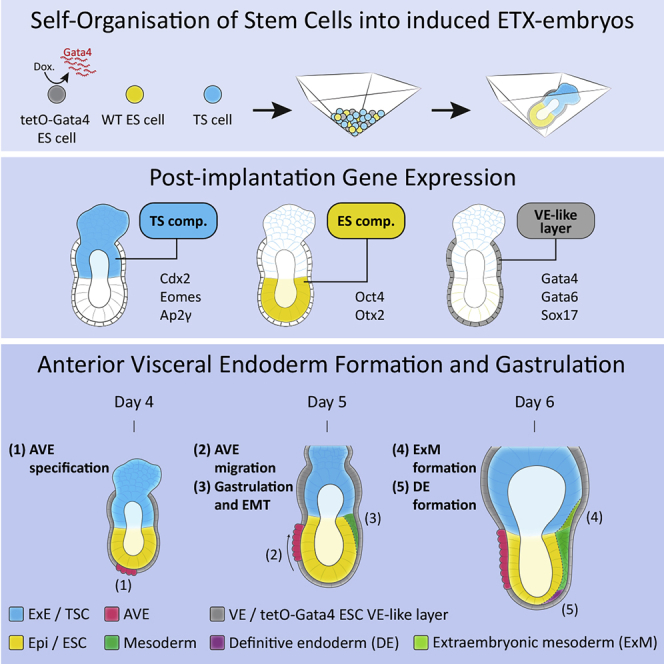

## Teaser

Amadei et al. have generated stem-cell-based structures that resemble mouse post-implantation embryos and have the potential to form the anterior-posterior axis and undergo gastrulation *in vitro*. Single-cell sequencing shows gene-expression patterns similar to those of the natural embryo at a comparable stage of development.

## Introduction

Mouse embryo development relies on interactions between the epiblast (EPI), the extra-embryonic ectoderm (ExE), and the visceral endoderm (VE), which are the respective precursors of the embryo proper, the placenta, and the yolk sac. These tissue interactions transform the embryo from the blastocyst into the egg cylinder: the EPI and the ExE polarize and open two luminal cavities, which, eventually, fuse to form the proamniotic cavity (Tam and Loebel, 2007; Bedzhov and Zernicka-Goetz, 2014; Christodoulou et al., 2018), while VE grows to envelop embryonic and extra-embryonic tissues (Christodoulou et al., 2019). The anterior-posterior axis is established by a group of VE cells that migrates unilaterally to protect anterior EPI from posteriorizing signals by secreting Dkk1, Cerl, and Lefty1, which antagonize Wnt, Bmp4, and Nodal signaling (Thomas et al., 1998; Weber et al., 1999; Stower and Srinivas, 2017). The anterior EPI later upregulates the expression of Sox1 and commits to become neuroectoderm and surface ectoderm (Bylund et al., 2003; Kan et al., 2004; Zhao et al., 2004). Mesodermal identity in the EPI is marked by the upregulation of Brachyury (Bry) at the EPI/ExE boundary, followed by the epithelial-to-mesenchymal transition of cells that egress through the primitive streak (PS) to form all three germ layers (Rivera-Pérez and Magnuson, 2005; Tam and Loebel, 2007).

Over recent years, stem cell lines derived from mouse embryonic and extra-embryonic tissues have become powerful tools to complement embryological studies (Evans and Kaufman, 1981; Tanaka et al., 1998; Kunath et al., 2005). Their ability to capture embryogenesis, however, is limited since each cell type is cultured in isolation and on their own do not acquire the morphology of embryos, hindering the modeling of the tissue-tissue interactions and signaling that are crucial for embryo patterning and morphogenesis *in vivo*.

To address this, we have developed a stem-cell-based model of embryonic development by combining ES, TS, and extra-embryo endoderm (XEN) stem cells into structures called ETX embryos (Sozen et al., 2018), and our results have been independently validated (Zhang et al., 2019). These ETX embryos closely resemble egg-cylinder-stage embryos at the morphological and transcriptional level. Yet, complex developmental events such as gastrulation occur only to a limited extent in this system, thus suggesting the need to improve it in order to capture the morphogenetic events occurring during natural embryo development.

### Design

To explain the limits of the ETX-embryo system, we reasoned that one or more cell types used for ETXembryo generation do not have the correct developmental potential to recapitulate embryo development. Indeed, XEN cells were reported to be more similar to parietal endoderm than to primitive endoderm (PrEn) or VE (Paca et al., 2012; Moerkamp et al., 2013), pointing to a need to replace XEN cells as a partner in the ETX embryo.

As induction of two transcription factors Gata4 or Gata6 in ESCs is sufficient to differentiate them toward endoderm (Shimosato et al., 2007; Schröter et al., 2015; Mathew et al., 2019), we hypothesized that such “induced” endodermal cells could functionally replace XEN cells. Here, we test this hypothesis by combining ES and TS cells with ESCs transiently expressing Gata4 in response to Doxycycline (Dox). The resulting *induced ETX embryos* (iETX embryos), in addition to expressing canonical post-implantation markers, can recapitulate complex morphogenetic events leading to formation and migration of the anterior signaling center and gastrulation.

## Results

### Induction of Gata4 in ES Cells Leads to Formation of Primitive Endoderm (PrEn) Lineage

**Figure 1:**
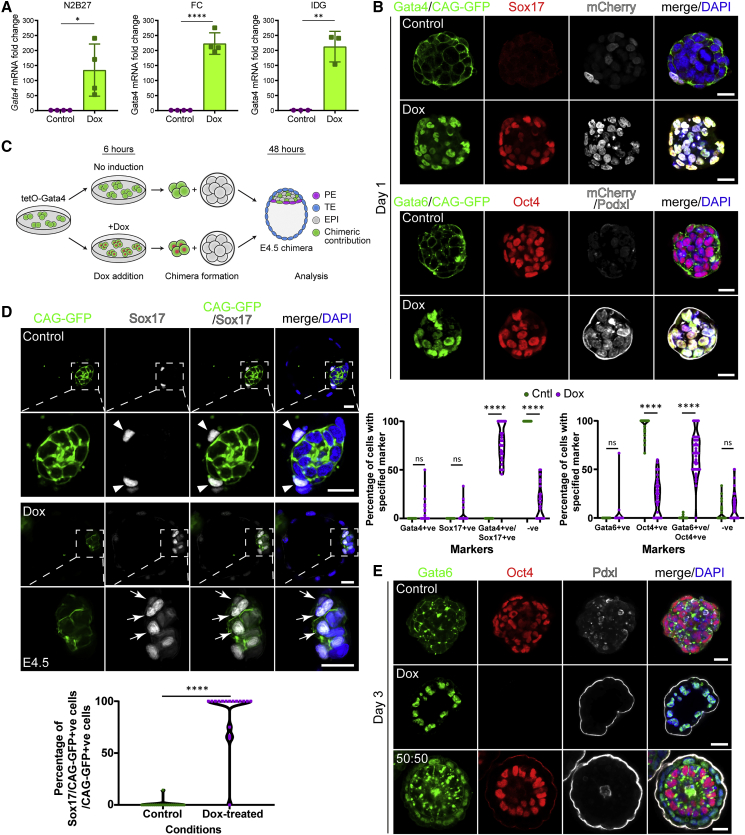
Treated CAG-tetOG4 ESCs Express Endodermal Markers and Contribute to Primitive Endoderm (PrEn) in Chimeras (A) *Gata4* mRNA expression in CAG-tetOG4 ESCs in N2B27 2iLIF (left, n = 4), FC 2iLIF (center, n = 4), and IDG 2iLIF (right, n = 3) after 6 h Dox treatment or control. Error bars, SD. (B) Top panel: CAG-tetOG4 ESC aggregates in control condition (top row) or Dox (bottom row), analyzed after 24 h for Gata4 (green, Alexa488), Sox17 (red, Alexa647), mCherry (gray), and DAPI (blue) (control, 49/49 structures; Dox, 37/37 structures; n = 3 each). Scale bar, 20 μm. Bottom panel: same as top, analyzed for Gata6 (green, Alexa488), Oct4 (red, Alexa647), mCherry and Podxl (gray, Alexa568), and DAPI (blue) (control, 49/52 structures; Dox, 45/45 structures; n = 3 each). Scale bar, 20 μm control and 15 μm Dox. Endogenous CAG-GFP in the green channel of control but downregulated in Dox (see Figure S1A). Below, quantification of the percentage of cells with the specified marker combinations in control and Dox aggregates. In the graphs, each dot is an aggregate. (C) Schematic of chimera aggregation: CAG-tetOG4 ESCs treated with Dox for 6 h or untreated and aggregated with E2.5 wild-type embryos. Contribution to either EPI or PrEn was assessed at E4.5. TE, trophectoderm. (D) Chimeras as in (C) analyzed for Sox17 (gray), CAG-GFP (green, with αGFP), and DAPI (blue). Contribution to PrEn with Dox-treated cells (bottom rows): 17/43 embryos from 3 females, 39%. Contribution to EPI of control cells (top rows): 22/22 embryos from 3 females. Scale and zoomed scale: 20 μm. Arrows, Sox17/CAG-GFP+ve cells; arrowhead, Sox17+ve/CAG-GFP-ve cells. In the graph, percentage of inner cell mass cells with PrEn identity was quantified. Each dot is an embryo. (E) CAG-tetOG4 ESC aggregates generated by combining cells either grown in control (top row), or Dox-treated cells for 6 hr (middle) or a 50:50 mixture of control and Dox-treated cells (bottom) and analyzed after 72 h for Gata6 (green), Oct4 (red), Podxl (gray), and DAPI (blue). Control: 41/43 aggregates, Dox: 55/56, 50:50: 60/65; n = 3 each. Scale bar, 20 μm. ^∗^p < 0.05,^∗∗^p < 0.01, ^∗∗∗∗^p < 0.0001, ns, nonsignificant. See also Figure S1.

To test whether replacing XEN cells with a cell type more similar to PrEn or VE could increase the developmental potential of ETX embryos, we modified our CAG-GFP/tetO-mCherry ES line (showing constitutive membrane GFP and transient mCherry expression following Dox-treatment) to transiently express Gata4 in response to Dox (CAG-GFP/tetO-mCherry/tetO-Gata4 ESCs, CAG-tetOG4 hereafter). We confirmed robust expression of *Gata4* mRNA after 6 h of Dox-treatment, independently of the ES culture medium (Figure 1A). Gata4 induction was necessary and sufficient for expression of endodermal proteins Gata4, Sox17, and Gata6 1 day after cell seeding, but Oct4 expression was retained at this time (Figure 1B); also note the CAG-GFP downregulation upon Gata4 induction, Figure S1A).

To test the effect of Gata4 overexpression on cell fate, we aggregated 8-cell stage embryos with ESCs and found that untreated CAG-tetOG4 ESCs contributed exclusively to the EPI, while Dox-treated CAG-tetOG4 ESCs could contribute to the PrEn (Figures 1C and 1D, 63 embryos). These results indicate that transient Gata4 expression is sufficient to change the potential of ESCs from EPI to PrEn lineage.

As an additional test to determine whether the CAG-tetOG4 ESCs could function as “building blocks” for ETX embryos, we generated ES aggregates using CAG-tetOG4 ESCs by combining (1) solely ESCs in control conditions (no Dox), (2) solely ESCs treated with Dox, or (3) Dox-treated and untreated ESCs in a 50:50 ratio and assessed cell fate after 3 days *in vitro*. In untreated aggregates, the ESCs maintained their identity and expressed Oct4 with a few exceptions (2/43 structures scored). We did not observe expression of Gata6 (Figure 1E), indicating that without Dox-treatment, upregulation of endodermal markers was extremely rare. Podxl distribution was scattered, indicating a lack of polarization and lumenogenesis (Shahbazi et al., 2017). In contrast, aggregates of Dox-treated ESCs completely downregulated Oct4 and induced Gata6 (55/56 structures scored). Podxl was on the outside of these aggregates in a continuous layer, suggesting that these structures were polarized but they did not undertake lumenogenesis (Figure 1E). Lack of Oct4 indicated that all cells failed to retain an ESC identity. Finally, aggregates generated by combining treated and untreated cells in a 50:50 ratio had an outer layer of Gata6-expressing cells and an inner compartment of Oct4-expressing ESCs. These aggregates underwent polarization and lumenogenesis and their morphology was reminiscent of the post-implantation EPI surrounded by VE (VE-like layer) (60/65 structures scored) (Figure 1E).

A lineage-tracing experiment with Dox-treated CAG-tetOG4 ESCs and unlabeled ESCs showed that the VE-like layer expressed CAG-GFP, confirming that it was generated by the Dox-treated cells with the Gata4 transgene (Figures S1B and S1C). These results suggest that combining Dox-treated and untreated CAG-tetOG4 ESCs leads to the generation of self-organising aggregates containing VE-like and EPI-like compartments, a crucial building block in ETX embryogenesis.

### iETX Embryos Self-Assemble and Express Canonical Markers of Post-implantation Embryos

**Figure 2:**
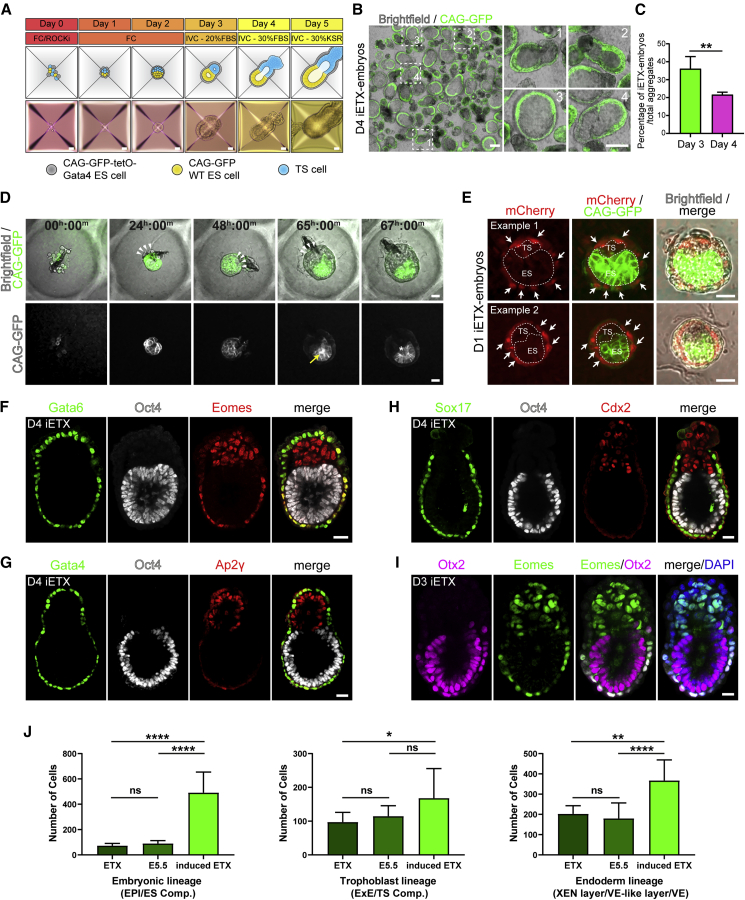
iETX Embryos Express Canonical Post-implantation Embryo Markers (A) Top: Time course and schematic of iETX embryo generation from cell seeding on day 0 to day 5. Bottom: Images of a representative iETX embryos at specific time points. Scale bar, 100 μm. (B) The contents of a single AggreWell were collected at day 4 to quantify formation efficiency of iETX embryos. ES compartment and VE-like layer express CAG-GFP; the TS compartment is unlabeled. Scale bar, 200 μm. Panels (right) highlight representative structures such as well-formed iETX embryos (1 and 2); an inflated iETX embryo (3); a well-formed iETX embryo in the process of inflating (4). Scale bar, 150 μm. (C) Quantification of iETX embryo formation at either 3 or 4 days. Day 3 = 1,542/4,187 structures (n = 4); day 4 = 960/4,410 structures (n = 4). Error bar: SD. (D) Time-lapse still images of iETX embryo formation over the course of 67 h. Top: The ES compartment and VE-like layer express CAG-GFP; TS compartment is unlabeled. Bottom, CAG-GFP ESCs alone (gray). White arrowheads highlight TS cells, yellow arrow and asterisk the forming ES lumen. Six examples. Scale bar, 30 μm. (E) iETX embryos at 1 day of development. ES and TS compartment are enclosed with a dashed line. Dox-treated CAG-tetOG4 ESCs transiently express mCherry and downregulate CAG-GFP (arrows). 91/109 structures from 2 independent experiments. Scale bar, 30 μm. (F–I) iETX embryo at day 4 stained for (F) Gata6 (green), Oct4 (gray), and Eomes (red) (41/42 structures). (G) Gata4 (green), Oct4 (gray), and Ap2γ (red) (18/19 structures). (H) iETX embryo at day 4 stained for Sox17 (green), Oct4 (gray), and Cdx2 (red) (21/21). (I) iETX embryo at day 3 stained for Otx2 (purple), Eomes (green), and DAPI (blue) (27/31). (F–H) n = 3 each; scale, 30 μm. (I) n = 3; scale bar, 20 μm. (J) Lineage quantification in iETX embryos in comparison with E5.5 and ETX embryos. 15 iETX, n = 3; 10 ETX and 10 E5.5 embryos, n = 3: from (Sozen et al., 2018). Error bars: SD. ^∗^p < 0.05,^∗∗^p < 0.01, ^∗∗∗∗^p < 0.0001, ns, nonsignificant. See also Figure S2.

To test whether XEN cells could be functionally replaced by CAG-tetOG4 ESCs to generate ETX embryos, we combined wild-type CAG-GFP ESCs, Dox-treated CAG-tetOG4 cells, and TS cells in AggreWells (Figure 2A). After 24 h, cells had aggregated but no clear organization could be discerned. After 48 h, aggregates had fully compacted and increased in size. At 72 h, aggregates began elongation and developed an egg-cylinder-like morphology reminiscent of ETX and natural post-implantation embryos (Sozen et al., 2018). At 96 and 120 h, the structures outgrew the microwells, requiring transfer to a bigger culture vessel. From 72-h onward, we could observe formation of the VE-like layer, encompassing the ES and the TS cells, which had formed distinct abutting compartments. Lumen formation in ES and TS cells was observed at 72 h, and at 96 h the lumens merged. Because these ETX embryos were generated by replacing XEN cells with Dox-treated “induced” CAG-tetOG4 cells, we termed them iETX embryos.

To calculate the formation efficiency of iETX embryos, we collected the whole contents of several wells 3 days and 4 days after cell seeding and counted the number of structures containing an epithelial, GFP-expressing ES compartment, segregated from an unlabeled TS compartment, both surrounded by a layer of cells, over the total number of structures (Figures 2B and S2A). At day 3, over 30% of the structures showed correct morphology, but at day 4, this fraction had decreased to 20% (Figure 2C), likely because some structures failed to develop properly between days 3 and 4 (Figure 2B). CAG-GFP expression was downregulated after Dox-treatment of the CAG-tetOG4 ESCs in the VE-like layer (Figure S2B), as in natural embryos (Bedzhov and Zernicka-Goetz, 2014). For the remaining experiments, we restricted our analyses to iETX embryos with correct morphology.

To follow iETX development, we seeded our cells in PEG-hydrogel plates to monitor iETX embryogenesis by live imaging (Figures 2D and S2C). After 24 h, cells compacted and formed an aggregate. Between 24 and 48 h, a CAG-GFP ES compartment became surrounded by a thin layer of cells; at 24 h the TS cells formed a very small layer or clump in the structure. The TS compartment emerged between 48 and 72 h, and the layer surrounding both ES and TS compartments became thicker and more prominent. The ES compartment underwent lumenogenesis between 60 to 67 h. At 72 h and onward the structure elongated until it grew larger than the diameter of the well. Similar to our observations with ETX embryos (Sozen et al., 2018), iETX embryos developed to strongly resemble mouse post-implantation embryos. The VE-like layer, unlike the ES compartment, was mCherry positive for the first 24 h, indicating that it had been generated by Dox-treated CAG-tetOG4 cells (Figures 2E and S2D). Transient mCherry disappeared by 72 h (Figure S2E).

To ascertain whether iETX embryos expressed the appropriate post-implantation markers, we analyzed canonical lineage markers 4 days after cell plating. The VE-like layer expressed VE markers Gata6, Gata4, and Sox17 (Figures 2F–2H and S2F); the TS compartment expressed the ExE markers Eomes, Ap2γ and Cdx2 (Figures 2F–2H and S2F); and the ES compartment expressed EPI markers Oct4 and Otx2 (Figures 2F–2H, 80/82 structures; Figure S2F). Finally, the VE-like layer adjacent to the ES compartment expressed Eomes and Otx2 in nearly all the cases examined (Figures 2F and 2I, 68/73; Figure S2F), in agreement with Eomes and Otx2 expression in the embryonic part of the VE. In comparison, the XEN layer of ETX embryos expressed Otx2 and Eomes in 40% of cases (Sozen et al., 2018). iETX embryos at day 4 had a higher number of cells in each lineage in comparison with ETX embryos at day 4 (Figure 2J).

### iETX Embryos Form the Anterior Signaling Center

**Figure 3:**
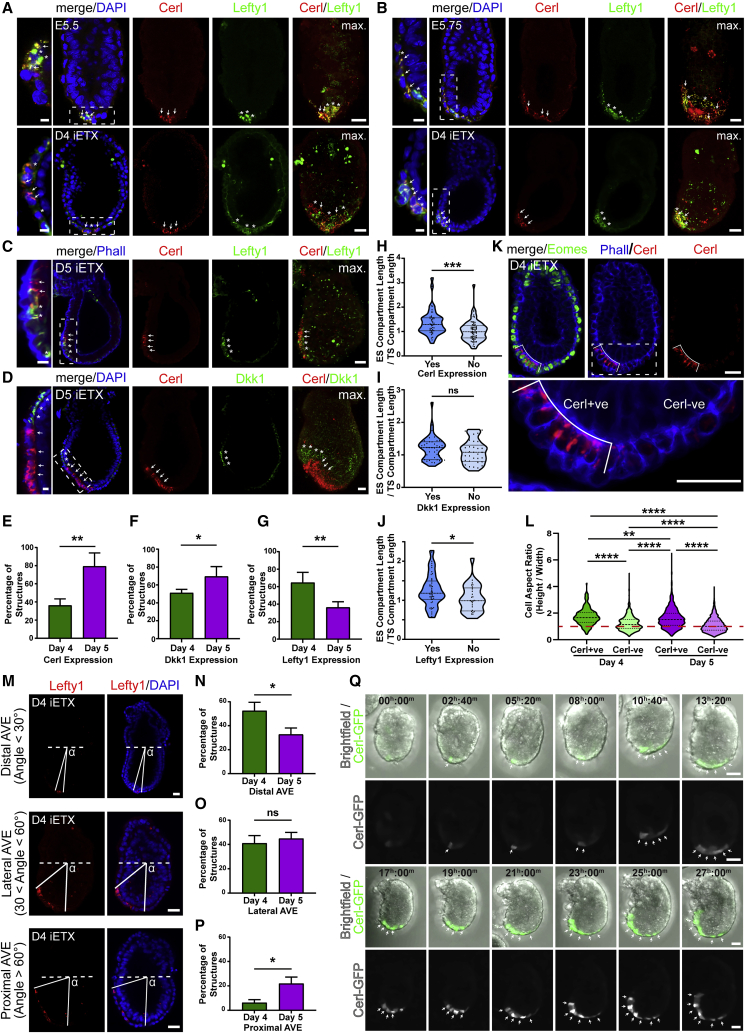
iETX Embryos Form a Migrating Anterior VE (AVE) (A and B) Wild-type embryos (top) at E5.5 (A) or E5.75 (B) and representative iETX embryo at day 4 (bottom) analyzed for Cerl (red, arrows), Lefty1 (green, asterisks), and DAPI (blue); max, maximum projection. Embryos: (A and B) 1 example each. iETX embryo: (A) 8/16 examples; (B) 8/16 examples, n = 3 each; scale bar, 20 μm. (C and D) Representative iETX embryo at day 5 analyzed for Cerl (red, arrows), Lefty1 (C, green, asterisks), Dkk1 (D, green, asterisks), and DAPI or Phalloidin (blue). (C) 23/63 structures. (D) 46/73 structures, n = 3 each; max., maximum projection; scale bar, 30 μm. (A–D) Dashed rectangles are magnified on the left. Scale bar, 10 μm. (E–G) Expression of Cerl (E), Dkk1 (F), and Lefty (G) at 4 and 5 days in iETX embryos (Cerl: 38/106 [day 4, n = 3] and 133/171 [day 5, n = 4]; Dkk1: 46/89 [day 4, n = 5] and 52/73 [day 5, n = 3]; and Lefty1: 58/89 [day 4, n = 3] and 32/87 [day 5, n = 4] structures). Error bars, SD. (H–J) Plot of ES/TS length ratio of iETX embryos at day 4 with or without Cerl (H), Dkk1 (I), and Lefty1. (J) Cerl: yes, 38; no, 68; n = 3. Dkk1: yes, 43; no, 34 structures; n = 5. Lefty1: yes, 47; no, 25; n = 3. (K) Representative iETX embryo at day 4 analyzed for Cerl (red), Phalloidin (blue) and Eomes (green). Rectangle below shows Cerl+ve and −ve domains. Scale bar, 30 μm. (L) Cell aspect ratio quantification of the Cerl+ve and Cerl−ve domain in iETX embryos at days 4 and 5. Day 4 = 31 iETX embryos, n = 3. Cerl+ve group: 206 cells; Cerl−ve group: 779 cells. Day 5 = 128 iETX embryos, n = 3; Cerl+ve domain: 1,656 cells; Cerl−ve domain: 3,856 cells. In all violin plots, median and quartiles are shown. Line at 1 separates cuboidal/columnar shape ratio. (M) iETX embryos at day 4 analyzed for Lefty1 (red) and DAPI (blue); the AVE angle was traced as the Lefty1+ve cell closest to the ES/TS boundary and the distal tip. Scale bar, 30 μm. (N−P) iETX embryos at 4 and 5 days scored according to the position of the AVE: distal (N), lateral (O), and proximal (P) as described in (M). (N) day 4: 67/142 n = 11. day 5: 79/217 n = 11. (O) day 4: 63/142 n = 11. day 5: 93/217 n = 11. (P) day 4: 12/142 n = 11. day 5: 45/217 n = 11. Error bars: SEM. (Q) Time-lapse stills of AVE formation and migration in a Cerl-GFP reporter iETX embryo filmed from day 3 of development. Top and third row: Cerl-GFP+ve cells are green, every other cell is gray. Second and bottom row: Cerl-GFP+ve cells in gray (arrows). For DVE induction, 34 structures, n = 3, 12/34: induction at the distal tip; 8/34: Cer-GFP was already present; 12/34: no GFP upregulation; 2/34: signal was not induced at the tip. For AVE migration, 32 structures, n = 3, 11/32: migration, 10/32: no migration. 11/32: no signal. Scale bar, 30 μm. ^∗^p < 0.05, ^∗∗^p < 0.01, ^∗∗∗^p < 0.001, ^∗∗∗∗^p < 0.0001, ns, nonsignificant. See also Figure S3.

Since ETX embryos have a limited ability to form the anterior VE (AVE) signaling center, we wondered whether iETX embryos could better capture this process. We found that iETX embryos at 4 days of development expressed three canonical AVE markers, Cerl, Lefty1, and Dkk1 (Figures 3A, 3B, S3A, and S3B). Cells expressing Cerl, Lefty1, and Dkk1 were either at the distal tip of the iETX embryo, as at the time of AVE formation in E5.5 embryos (Figures 3A and S3A) or were positioned asymmetrically on one side of the iETX embryo, like their expression at E5.75 as the AVE begins its migration toward the future anterior (Figures 3B and S3B).

Expression of these markers was maintained at day 5 (Figures 3C and 3D). At day 4, 65% of iETX embryos expressed Lefty1, 50% expressed Dkk1, and 36% expressed Cerl (Figures 3E–3G, Cerl, 38/106; Lefty1, 58/89; Dkk1, 46/89). At day 5, 80% of iETX embryos expressed Cerl (Figure 3E, 133/171) and Dkk1 expression increased to 70% (Figure 3F, 52/73). In contrast, the proportion of iETX embryos expressing Lefty1 decreased from 65% to 40% between days 4 and 5 (Figure 3G, 32/87). We hypothesized that these differences at day 4 might relate to differences in the size of the iETX embryos, because Cerl is repressed by BMP4 signaling from the ExE (Rodriguez et al., 2005; Richardson et al., 2006; Soares et al., 2008), and Cerl is expressed only when the EPI has extended beyond a specific length (Mesnard et al., 2006). We, therefore, measured the length of the ES and TS compartments and correlated it to Cerl expression. iETX embryos with an ES compartment longer than the TS compartment consistently expressed Cerl; in contrast, when the TS compartment was longer than the ES compartment, there was no Cerl expression (Figure 3H). We did not observe this relationship for Dkk1 (Figure 3I) and it was much weaker for Lefty1 (Figure 3J), suggesting that Cerl expression might be regulated differently than Lefty1 and Dkk1. Analysis of the aspect ratio of Cerl-positive and Cerl-negative cells indicated that Cerl-positive cells were very similar in their morphology to AVE cells in embryos (Stower and Srinivas, 2017) (Figures 3K and 3L).

In embryos, the distribution of Cerl-, Lefty1-, and Dkk1-expressing cells varies between E5.5 and E6.0 because the AVE migrates toward the EPI/ExE boundary. At day 4, the AVE was either distal or lateral (52% and 41% of the cases, Figures 3N and 3O) and very rarely proximal (6%, Figure 3P). In contrast, at day 5, 22% of iETX embryos had proximal AVE and 33% had distal AVE, but there was no change in lateral AVE (Figures 3N–3P). Thus, at day 4, iETX embryos resemble E5.5/E5.75 embryos in terms of AVE specification and position. At day 5, the increase in the proportion of proximal AVE could either suggest AVE migration or localized *de novo* upregulation of AVE markers instead of cell migration.

To distinguish between these possibilities and investigate the dynamics of AVE induction and migration, we generated iETX embryos with a Cerl-GFP reporter/tetO-Gata4 line derived from our transgenic Cerl-GFP mouse line (Mesnard et al., 2004), in which GFP expression is under the control of the Cerl promoter. We began live imaging at day 3, when the reporter was not yet active (Figures 3Q and S3C). Reporter expression commenced in a single cell at the tip, expanded from a single cell to several, and initially resulted in the formation of a GFP-positive domain. Following consolidation of GFP expression at the iETX embryo’s tip, the AVE began its migration until it reached the boundary with the TS compartment after an average of 18 h (Figures 3Q, S3D, and S3E; Videos S1 and S2), which is slower than AVE migration in the embryo (Stower and Srinivas, 2017).

Video S1. iETX Embryo Live Imaging of AVE Migration, Related to Figure 3

Video S2. iETX Embryo Live Imaging of AVE Migration, Related to Figures 3 and S3

Tracking the migration trajectories of individual Cerl-GFP cells showed they migrated directionally toward the proximal end of iETX embryos (Figure S3F; Videos S3 and S4), consistent with AVE migration in embryos. When compared with the speed of AVE cells in embryos (0.23 ± 0.07 μm/min; mean ± SD) (Omelchenko et al., 2014), AVE in iETX embryos migrated at a similar albeit slightly reduced speed (0.198 ± 0.040 μm/min, 26 cells from 7 iETX embryos). Immunofluorescence confirmed the asymmetric localization of the Cerl-GFP cells in the majority (26/36) of iETX embryos examined (Figures S3G and S3H). This is an important improvement over the ETX embryos, in which we could observe neither the Cerl and Dkk1 proteins nor the AVE migration.

Video S3. iETX Embryo AVE Migration with Tracks, Related to Figures 3 and S3

Video S4. iETX Embryo AVE Migration with Tracks, Related to Figures 3 and S3

### iETX Embryos Specify Anterior and Posterior Domains

**Figure 4:**
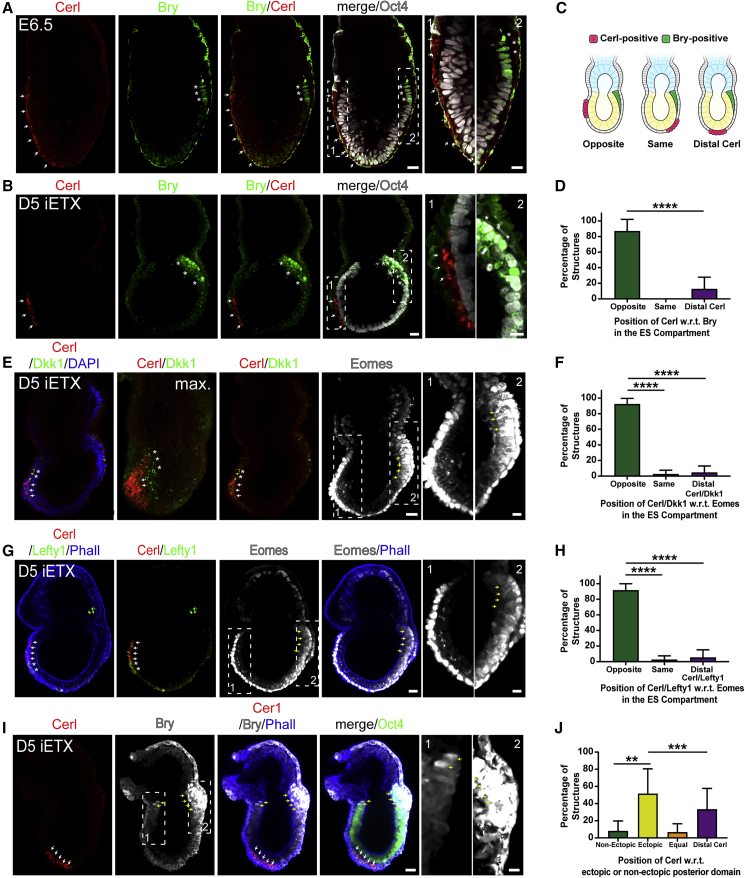
iETX Embryos Specify Anterior and Posterior Domains on Opposite Sides (A) E6.5 embryo with Cerl (red, arrows), Bry (green, asterisks), and Oct4 (gray). Natural embryo: 2 examples. (B) iETX embryo at day 5 analyzed for Cerl (red, arrows), Bry (green, asterisks), and Oct4 (gray). iETX embryos: 38/69 structures, n = 4. Scale bar, 30 μm. (C) Schematic of possible Bry/Cerl position combinations: opposite sides, same side, or Cerl is at the distal tip. (D) Scoring iETX embryo at 5 days of development according to (C). Opposite = 33 structures, same side = 0, distal = 4, n = 4. (E) iETX embryo at day 5 analyzed for Cerl (red, white arrows), Dkk1 (green, asterisk), Eomes (gray, yellow arrows), and DAPI (blue); max., maximum projection; 29/69, n = 3. Scale bar, 30 μm. (F) Scoring iETX embryos at day 5 according to the position of Cerl+ve/Dkk1+ve domain in relation to Eomes. Opposite = 28, same side = 1, distal = 2, n = 3. (G) Same as (E) but with Lefty1 (green, asterisks) and Phalloidin (blue). 18/54 examples, n = 3. Scale bar, 30 μm. (H) Same as (F) but with Cerl/Lefty1 in relation to Eomes. Opposite = 19, same side = 1, distal = 1, n = 3. (I) Representative iETX embryo at day 5 with ectopic Bry and analyzed for Cerl (red, white arrows), Bry (gray, yellow arrows), Oct4 (green), and Phalloidin (blue). Scale bar, 30 μm. 11/31, n = 4. (J) Scoring iETX embryo at 5 days according to the position of Cerl in relation to the posterior domain (Bry or Eomes) as described in Figure S4D. Non-ectopic = 5, ectopic = 28, equal sides = 3, distal Cerl = 25, n = 8. (A, B, E, G, and I) Dashed rectangles are magnified on the right. Scale bar, 10 μm. (D, F, H, J) Error bars, SD. ^∗∗^p < 0.01, ^∗∗∗^p < 0.001, ^∗∗∗∗^p < 0.0001. See also Figure S4.

We next sought to determine whether the formation of the anterior domain was accompanied by the establishment of a posterior one. Embryos at E6.5 express Cerl and mesoderm marker Bry (Wilkinson et al., 1990; Tosic et al., 2019) on opposite sides (Figures 4A and S4A). iETX embryos displaying Bry on only one side of the ES compartment on the fifth day of development comprised 55% of the total Bry- and Cerl-expressing structures (Figures 4B and S4B, 38/69 structures). In this subset, 87% of iETX embryos expressed Cerl on the side opposite to Bry (Figures 4C and 4D), whereas in 13% of iETX embryos, Cerl was at the distal tip. Importantly, we could not observe any instance in which Cerl and Bry were expressed on the same side (Figures 4C and 4D). We examined the expression of two other anterior markers, Dkk1 and Lefty1, in relation to Eomes, which is expressed in the PS (Tosic et al., 2019) and in iETX embryos at day 5 (Figure S4C), which confirmed that formation of anterior and posterior domains occur on opposite sides in 80%–90% (Cerl/Dkk1, 28/31; Cerl/Lefty1, 19/21) of the iETX embryos examined (Figures 4E–4H).

Formation of anterior and posterior domains on opposite sides was also conserved in iETX embryos expressing Bry around the ES/TS boundary (45%, 31/69)(Figure 4I). In most cases, one posterior domain was more expanded than the other, hence we considered the more expanded domain as the “true” posterior and the less expanded one as an ectopic posterior. In 50% of cases, the Cerl domain was on the side of the less expanded, ectopic posterior domain and opposite the large domain (Figures S4D and 4J); in only 8% of cases was the Cerl domain on the same side of the more expanded domain; in 7% of cases the posterior domains were equal and, thus, the position of the Cerl domain could not be assigned; and in 33% of the cases, the Cerl domain was distal (Figures S4D and 4J).

To determine whether the position and the size of the AVE could influence the likelihood that an iETX embryo would develop an ectopic posterior domain, we measured the angle of displacement of the Cerl domain away from the distal tip and toward the ES/TS boundary and the angle of extension of the Cerl domain. Structures without an ectopic Bry domain had a more extended Cerl domain, which was closer to the ES/TS boundary than structures with an ectopic Bry domain (Figures S4E and S4F). Location of the Lefty1 domain was not significantly different, but its extension was (Figures S4G and S4H). On the other hand, the Dkk1 domain was significantly closer to the ES/TS boundary in structures without ectopic Bry but the extension of the domain itself was no different (Figures S4I and S4J). These results suggest that the position and extension of the Cerl domain are important factors in preventing the expression of ectopic Bry in iETX embryos, whereas Lefty1 and Dkk1 may contribute to a lesser extent.

Out of the iETX embryos without Cerl, 65% displayed ectopic expression of Bry, suggesting that a subset of iETX embryos can break symmetry without Cerl (Figures S4K and S4L). Since a decrease in Nodal levels can rescue ectopic Bry expression in the absence of AVE (Perea-Gomez et al., 2002), we tested whether altering Nodal signaling levels can affect mesoderm formation. Culturing iETX embryos from day 4 to day 5 in Nodal inhibitor completely abrogated expression of Bry and Eomes (Figures S4M and S4N) in agreement with what was reported in natural embryos (Brennan et al., 2001). These results indicate that establishment of anterior and posterior domains occurs on opposite sides of the ES compartment in iETX embryos and Nodal is involved in this process as in natural embryos.

### iETX Embryos Undergo Epithelial-to-Mesenchymal Transition and Gastrulation

**Figure 5:**
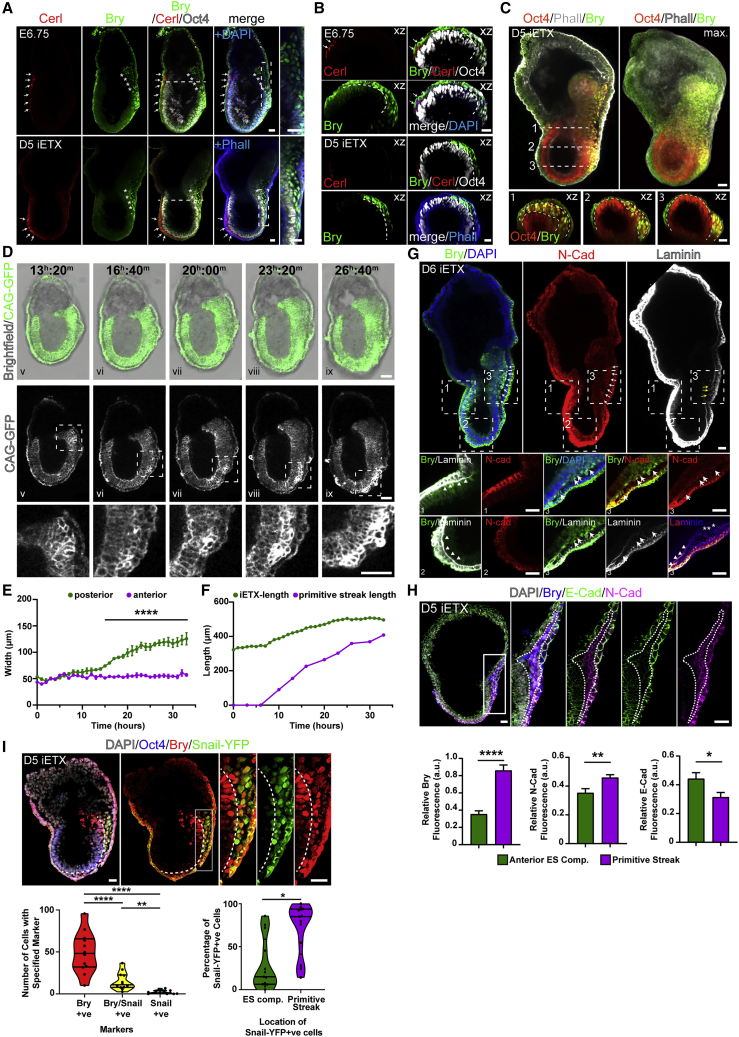
iETX Embryos Undergo EMT and Gastrulation at Day 5 (A) (Top) E6.75 mouse embryo (1 example) and (bottom) iETX embryo at day 5 (21/31 examples, n = 3) analyzed for Cerl (red, arrows), Bry (green, asterisks), Oct4 (gray), and DAPI (embryo, blue) or Phalloidin (iETX embryo, blue). Rectangles are on the right. Scale bar, 30 μm. (B) Orthogonal sections along the dashed lines in (A) to show mesodermal wings. Markers are same as (A). Scale bar, 30 μm. (C) iETX embryo at 5 days of development analyzed for Bry (green), Oct4 (red), and Phalloidin (gray). Orthogonal sections in the xz from indicated dashed lines are at the bottom (1, 2, 3) to highlight mesodermal wings and EMT in the structure (28 examples, n = 3). Scale bar, 30 μm. (D) Time-lapse stills of an iETX embryo imaged from day 4 to day 5. Top row: TS cells are in gray, wild-type CAG-GFP and induced CAG-tetOG4 are in green. Center row: CAG-GFP cells are displayed in gray. Dashed squares are magnified at the bottom and highlight the prospective posterior side. 6/13 structures with comparable EMT, n = 3. Scale bar, 50 μm. (E) Measurement of the thickness of the anterior, nongastrulating side (purple dots), and the posterior, gastrulating side (green dots) over time in the iETX embryo in (D). Error bars, SD. (F) Measurement of iETX embryo length (green dots) and extending PS length over time for the iETX embryo in (D). (G) iETX embryo at day 6 analyzed for Bry (green), N-Cad (red), laminin (gray), and DAPI (blue). PS (white arrows) and breached basement membrane (yellow arrows) at the posterior are highlighted. Squares below highlight (3) the gastrulating posterior, analyzed with Bry (green), N-Cad (red) and laminin (gray), and laminin (fire). Arrows: Bry/N−Cad+ve cells undergoing EMT; arrowheads: intact laminin; asterisks: ruptured laminin tract. Panels (1) and (2) show anterior and distal tip without N-Cad and with intact laminin tract (arrowheads). 21/24 examples, n = 4. Scale bar, 30 μm. (H) iETX embryo at day 5 analyzed for DAPI (gray), Bry (blue), E-Cad (green), and N-Cad (magenta); rectangle is shown on the right. Scale bar, 30 μm. Below, quantification of Bry, E-Cad, and N-Cad expression in the anterior EPI and PS. Mean fluorescent intensities of the markers were normalized to DAPI intensity. 20 examples, n = 3. Error bars, SEM. (I) iETX embryo at day 5 generated with a Snail-YFP reporter line and analyzed for DAPI (gray), Oct4 (blue), Bry (red), and Snail-YFP (green, αGFP). Dashed line marks the PS. White rectangle is on the right. Scale bar, 30 μm. 27/42, n = 3. Bottom, left: quantification of Bry+ve, Snail/Bry+ve, and Snail+ve cells in iETX embryos. Each dot is an iETX embryo. Bottom, right: percentage of Snail+ve cells found in the ES comp. or PS. Each dot is an iETX embryo, 13 examples, n = 3. In all violin plots, median and quartiles are shown. ^∗^p < 0.05, ^∗∗^p < 0.01, ^∗∗∗∗^p < 0.0001, ns, nonsignificant. See also Figure S5.

Having observed robust formation of anterior and posterior domains in the iETX embryo, we wished to determine whether the posterior side could also establish a PS to undertake gastrulation. Gastrulation is evident in embryos at E6.75 when Bry-positive cells near the EPI/ExE boundary undergo the epithelial-to-mesenchymal transition (EMT) and egress to form a mesoderm layer between the EPI and the VE. We found that iETX embryos also developed Bry-expressing cells between the ES compartment and the VE-like layer between days 5 and 6 (Figures 5A–5C) but not on the opposite side of the structure where Cerl was expressed.

To understand the dynamics of this process, we performed time-lapse imaging of iETX embryos from day 4 (Figures 5D and S5A; Video S5). At the onset of imaging, cells on all sides of the GFP-expressing ES compartment were epithelial and columnar, but at 13 h, the cells on one side of the ES and TS compartment boundary began to change their shape from columnar to rounded and positioned themselves between the ES compartment and the VE-like layer (Figure 5D). Between 13 and 16 h, the layer formed by the egressing cells had almost extended to the distal tip, similar to PS formation in embryos (Arnold and Robertson, 2009). Throughout this process, the opposite, putative anterior side remained epithelial and did not undergo EMT. PS extension continued until 27 h, with the streak reaching the distal tip of the iETX embryo (Figure 5D). During gastrulation, the thickness of the posterior side progressively increased relative to the anterior side, which remained constant and the streak steadily increased in length (Figures 5E and 5F).

Video S5. Live Imaging of iETX Embryo Gastrulation, Related to Figures 5 and S5

On day 6, iETX embryos showed a large, Bry-positive domain on the side of the ES compartment undertaking gastrulation (Figure 5G). In most of the structures examined, 40% of Bry-positive cells expressed N-cadherin (Figure S5B), and there was downregulation of laminin in the basement membrane at the point of EMT, in contrast to other parts of the iETX embryo not undergoing EMT (Figures 5G and S5C). Egressed cells were Bry-positive and expressed laminin; formation of a laminin layer could be detected between the mesoderm and the VE-like layer (Figure 5G). The PS of iETX embryos expressed higher levels of Bry (Figure 5H), which was accompanied by downregulation of E-cadherin and upregulation of N-cadherin (Figure 5H). Finally, egressed cells co-expressed Bry and the EMT marker Snail (Figure 5I), suggesting that gastrulation in iETX embryos follows the same process as the natural embryo (Ramkumar et al., 2016; Punovuori et al., 2019; Kyprianou et al., 2020). Based on Snail-YFP expression, 64% of the structures were undergoing EMT at day 5 (27/42, see also Figure S5D).

Gastrulation sees not only the formation of embryonic mesoderm but also the proximal migration of mesodermal cells to form extra-embryonic mesoderm (Sutherland, 2016; Saykali et al., 2019). In 45% of day 5 iETX embryos undergoing EMT, Bry/Oct4-expressing cells were observed between the VE-like layer and the TS compartment (Figures S5E and S5F) and either formed patches above the ES/TS boundary or were arranged as a line of cells between the TS compartment and the VE-like layer overlaying the TS compartment (Figure S5F, orange asterisks). In 20% of the examined iETX embryos, Bry was co-expressed with Runx1, which marks extra-embryonic mesoderm cells committing to the hematopoietic lineage (Tanaka et al., 2014) (Figure S5G). This represents an important improvement over ETX embryos, in which we did not observe formation of extra-embryonic mesoderm.

### iETX Embryos Generate Heterogeneity in the Primitive Streak and Form Definitive Endoderm

**Figure 6:**
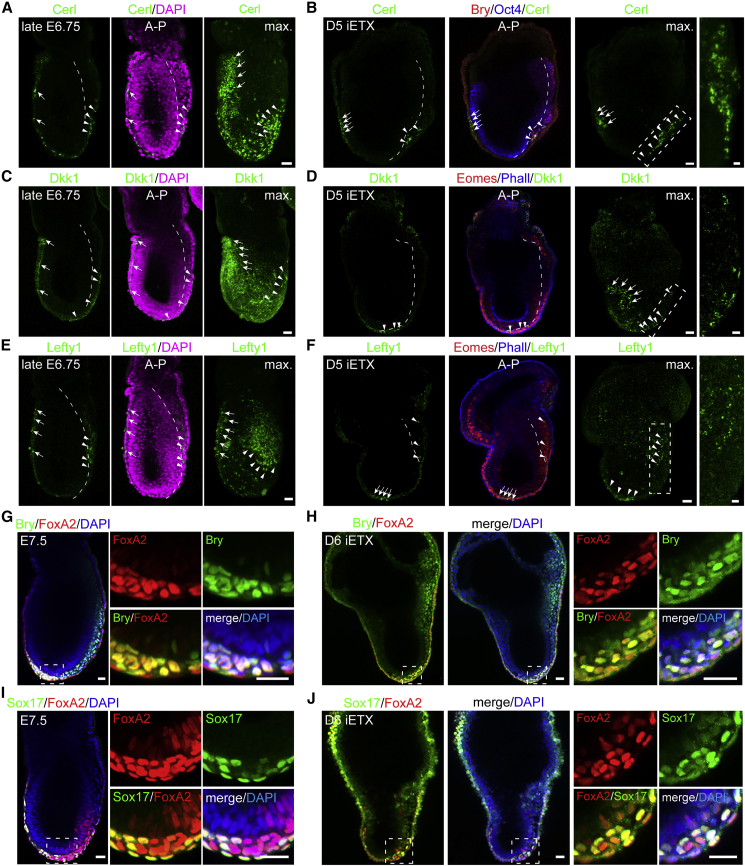
iETX Embryos Generate Heterogeneity in the Primitive Streak and Form Definitive Endoderm (A, C, and E) Natural embryos at late E6.75 analyzed for DAPI (magenta) and (A) Cerl (green), (C) Dkk1 (green), and (E) Lefty1 (green). The maximum projection of Cerl, Dkk1, and Lefty1 is in the right panels. Dashed line marks the PS. (Cerl, Dkk1, and Lefty1: 3 embryo examples each) Scale bar, 30 μm. (B, D, and F) iETX embryos collected at 5 days of development and analyzed for (B) Bry (red), (D and F) Eomes (red) and (B) Cerl (green), (D) Dkk1 (green), and (F) Lefty1 (green), (B) Oct4 (blue), and (D and F) Phalloidin (blue). Cerl, Dkk1, and Lefty1 maximum projection is in the right panels. Dashed rectangles are magnified on the right. (Cerl: 17/33, n = 3; Dkk1: 21/56, n = 3; Lefty1: 27/34, n = 3) (A–F): white arrows indicate the AVE and white arrowheads posterior expression of the AVE marker; (A–P) indicates the anterior-posterior axis. Scale bar, 30 μm. Cerl, Lefty1 and Dkk1 in these panels were visualized using Alexa-568 or Alexa-647 secondary. Dashed rectangles scale bar, 10 μm. (G and H) E7.5 mouse embryo (G) and iETX embryo at day 6 of development (H) analyzed for FoxA2 (red), Bry (green), and DAPI (blue); dashed squares are magnified on the right (Embryo: 2 examples; iETX embryo: correct patterning: 45/52; extended streak: 37/52, n = 6). Scale bar, 30 μm. (I and J) E7.5 mouse embryo (I) and iETX embryo at day 6 of development (J) analyzed for FoxA2 (red), Sox17 (green), and DAPI (blue); dashed squares are on the right (Embryo: 3 examples; iETX embryo: extended streak with Sox17/FoxA2 in 17/31 examples, n = 3). Scale bar, 30 μm. See also Figure S6 and Table S1.

During gastrulation, the developing streak generates multiple mesodermal and endodermal cell types required for subsequent development and secretes Cerl, Lefty1, and Dkk1 (Robb and Tam, 2004) leading us to ask whether similar changes take place in gastrulating iETX embryos. Analysis of gastrulating iETX embryos at day 5 indicated that, similar to natural embryos (Figures 6A, 6C, and 6E), we identified expression of Cerl, Dkk1, and Lefty1 in the AVE and also in the developing PS (Figures 6B, 6D, and 6F). Posterior expression of Cerl, Lefty1, and Dkk1, respectively, was present in 51% (17/33), 79% (27/34), and 37% (21/56) of the examined iETX embryos, indicating gastrulation processes further than in ETX embryos.

We also observed co-expression of Bry and FoxA2 at day 6 in the distal part of the PS (71%, 37/52) similar to E7.5 natural embryos (Figures 6G and 6H), which is indicative of axial mesoderm formation, and FoxA2 and Sox17 expression, indicative of definitive endoderm (Figures 6I and 6J) (Nowotschin et al., 2019). Bry and Sox17 were co-expressed in the ES compartment, suggesting that a subset of cells express mesodermal markers before acquiring definitive endoderm identity (Figure S6A), similar to the embryo (Nowotschin et al., 2019).

We could not determine whether expression of neuroectoderm markers would occur, because after day 6, iETX embryos became dark and lost their cylindrical appearance, suggesting that 6 days of culture is the current limit for our system. Developmental milestones of iETX embryos are presented in Figure S6B and Table S1.

**Figure 7:**
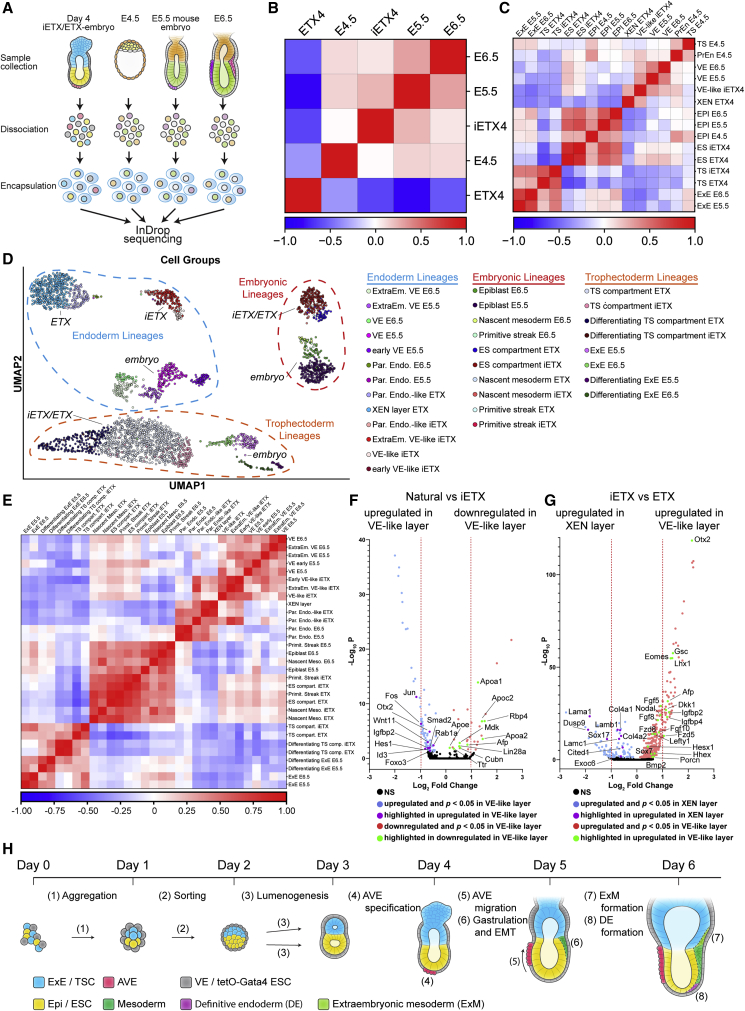
The VE-like Layer Formed by CAG-tetOG4 ES Cells Is More Similar to Natural VE (A) Schematic of inDrop sequencing. (B) Global correlation matrix of natural embryos (E4.5, E5.5, and E6.5) and ETX and iETX embryos (day 4). (C) Correlation matrix with the same samples in (B) subdivided according to their lineage. Natural samples: EPI, ExE, VE, and PrEn; ETX embryos: embryonic stem cell compartment (ES comp), trophoblast stem cell compartment (TS comp), extra-embryonic endoderm stem cell layer (XEN layer), and tetoG4 cell-derived layer (VE-like layer). (D) Single-cell sequencing UMAP and sample separation in subpopulations based on sample type (natural versus ETX), age (E5.5, E6.5, and day 4) and developmental stage. Developmental stage classification was based on leiden clusters and the markers in Figure S7A. (E) Correlation matrix of the single-cell subpopulations in (D). (F and G) Volcano plot of genes downregulated or upregulated in the VE-like layer of iETX embryos versus natural VE (F) or of genes upregulated in the VE-like layer of iETX embryos versus upregulated in the XEN layer of ETX embryos (G). (H) Summary: Dox-treated, induced tetOG4 ESCs combined with wild-type ES and TS cells aggregate and sort to generate structures organized into discreet ES and TS compartments surrounded by a VE-like layer, called induced ETX embryos. iETX embryos open a lumen between day 2 and day 3 and establish the AVE at day 4, which then migrates between day 4 and day 5 to establish the anterior domain. On the opposite side at day 5, Bry and Eomes expression in the ES compartment indicates formation of the posterior domain. On day 5 and onward, the posterior domain undergoes EMT and gastrulation, forming embryonic and extra-embryonic mesoderm and definitive endoderm. See also Figure 7; Table S2.

To understand further the developmental potential of iETX embryos, we performed inDrop single-cell RNA sequencing on iETX embryos and ETX embryos at day 4 and compared them with embryos at E4.5(GEO: GSE134240, Sozen et al., 2019), E5.5, and E6.5 (Figure 7A). Pearson correlation coefficients between all samples indicated that iETX embryos were most similar to E5.5 natural embryos (Figure 7B). We subdivided the natural and ETX/iETX embryos into their constitutive lineages and observed that all TS cells clustered with ExE derivatives and all ESCs clustered with EPI derivatives, irrespective of whether they originated from ETX or iETX embryos. On the other hand, VE, XEN layer, and VE-like layer correlated less. The XEN layer showed some similarity with the PrEn of the E4.5 embryo but correlated little with E5.5 and E6.5 VE; the VE-like layer of iETX embryos showed similarities to E4.5 PrEn and to the VE at E6.5 but was most similar to E5.5 VE. This suggests that tetOG4 ESCs may generate an endodermal layer more similar to VE (Figure 7C).

To identify specific cell subpopulations in this dataset, gene signatures (Figure S7A) and Leiden clustering were utilized for further classifications. The ExE cluster cells were divided into ExE or differentiating ExE based on reciprocal expression of the TS marker Cdx2 and differentiation marker Gata2, and we also observed these signatures in the TS compartment. The EPI cluster expressed the primed pluripotency marker Otx2; the E6.5 EPI could be subdivided into nascent mesoderm, based on Mesp1 expression, or into a PS subpopulation, based on Eomes and Nanog (Saga et al., 1996; Pijuan-Sala et al., 2019). Nascent mesoderm and PS signatures were already present in the ES compartment, suggesting that it is primed for gastrulation and may be at a more advanced stage than the E5.5 EPI. Nevertheless, expression of the EMT marker Snail1 mostly in the E6.5 EPI and not in the ES compartment confirmed that EMT in iETX embryos does not occur until day 5. The VE cluster was initially identified by Gata4 expression and then subdivided into parietal endoderm based on LamB1 expression, in embryonic VE based on Otx2/Eomes expression, and cells that were Gata4-positive, but LamB1/Otx2/Eomes-negative were classified as extra-embryonic VE. Within the embryonic VE cluster, we identified cells expressing Cerl, Lefty1, Hhex, and Dkk1, indicating the AVE. In iETX embryos, AVE-like cells expressed Dkk1, Lefty1, and Hhex1 but low Cerl, suggesting an earlier stage of AVE development, more similar to E5.25 (Hoshino et al., 2015). We could detect an additional, small subpopulation of cells expressing Hhex, Lefty1, and low Otx2, similar to the expression of these markers in the E4.5 PrEn (Hoshino et al., 2015), we termed these “early VE-like layer” (Figure 7D).

With these classifications, TS cells from ETX and iETX embryos clustered together with ExE as a group, although differentiating TS cells were most different from ExE at E5.5 and E6.5. ESCs from the ETX and iETX embryos clustered together with EPI. Subdivision of the endodermal lineages into these subpopulations shed further light on the relationship between the XEN layer, the VE-like layer, and the VE. The XEN layer samples were most similar to the parietal endoderm of embryos. By contrast, the VE-like layer correlated with the VE and differed from E5.5 and E6.5 parietal endoderm. This suggests that while induced ESCs are not identical to the natural VE, they are a marked improvement over standard XEN cells in recapitulating the natural gene-expression signature (Figure 7E).

Differential gene-expression analysis using Wilcoxon rank sum test between the VE-like layer and the VE indicated that 25 genes were downregulated and 71 genes were expressed at higher levels in the VE-like layer of iETX embryos in comparison with the VE (Figure 7F). Genes with higher expression in iETX embryos included Smad2, Wnt11, and Id3, suggesting differences in Nodal, Wnt, and Bmp signaling, and Igfbp2, which may indicate differences in metabolic pathways. Comparison of the VE-like layer to the XEN layer (Figure 7G) revealed that the former expressed several regulators of AVE and embryonic VE formation, including Hhex, Hexs1, Gsc, Lhx1, and Afp. Consistent with our previous observations, we also detected Eomes, Lefty1, and Dkk1. The VE-like layer expressed a higher level of Nodal than the XEN layer. Gene ontology analysis of the genes upregulated in the VE-like layer of iETX embryos also indicated the presence of regulators of the Wnt pathway (Figures S7B–S7E; Table S2). Comparison of the VE with the XEN layer of ETX embryos showed differences in the same genes identified when we compared the VE-like layer with the XEN layer (Figures S7F–S7H), once again indicating the high degree of similarity between VE and VE-like layer. In summary, the VE-like layer of iETX embryos expresses a number of crucial regulators of VE identity, AVE specification, as well as factors regulating mesoderm formation and gastrulation, which could increase the developmental potential of iETX embryos (Figure 7H).

## Discussion

Stem cell models of embryo development hold the promise to streamline the study of uncharacterized genes, novel drugs, and developmental pathways because of their modular design and ease of separately manipulating each compartment (Brassard and Lutolf, 2019). Here, we have developed a model of mouse embryo development using iETX embryos, which builds upon, and significantly improves, our previously reported ETX embryos (Sozen et al., 2018).

Morphologically, iETX embryos are very similar at days 4 and 5 to natural embryos at E5.5 and E6.5 and express the canonical markers of post-implantation development at these stages. Importantly, iETX embryos can induce formation of AVE, as indicated by the expression of Cerl, Lefty1, and Dkk1, and this AVE is able to migrate asymmetrically toward the boundary of ES- and TS-derived compartments. Anterior and posterior domains are specified on opposite sides and the embryo-like structure then undertakes gastrulation with formation of embryonic and extra-embryonic mesoderm and definitive endoderm. These processes occur reproducibly in the great majority of the structures examined and make this a valuable system to study AVE induction and gastrulation *in vitro*.

At a technical level, iETX embryos are easier to generate because they grow in standard, nonconditioned tissue-culture media and do not require the establishment of novel XEN lines, but existing ES lines can be modified for this assay. Our single-cell sequencing data show that replacing XEN cells with tetOG4 ESCs is beneficial because they make a VE-like layer, which is more similar to the natural VE and expresses several regulators of AVE development, potentially accounting for the broader developmental potential of iETX embryos.

### Limitations

One current limitation is the observed downregulation of Lefty1 at day 5. Since Lefty1 is a direct Nodal target (Takaoka et al., 2017), this may mean that Nodal signaling is not sustained enough at that point. Our data also suggest that expression of Cerl, Lefty1, and Dkk1 in the AVE may be differentially regulated, but this needs to be tested in the future. Second, when the AVE is not positioned correctly, posteriorizing signals from the TS compartment induce mesoderm formation on both sides of the ES compartment. One of the two sides is consistently more advanced than the other, suggesting that even incomplete migration of the AVE is sufficient to set up an asymmetry that favors one posterior domain over the other. Finally, iETX embryos cannot be cultured beyond 6 days; thus, improved culture conditions will be needed to test their developmental potential further.

We conclude that the state of the VE precursor cells plays a considerable part in defining the extent of development of synthetic embryo structures. Here, by expressing Gata4 in ESCs, we generate VE-like cells that are able to undertake considerably more extensive AVE development and PS formation at gastrulation in iETX embryos and, therefore, provide a better building block for these post-implantation structures. We show that this is a consequence of the greater similarity of these cells to natural VE than XEN cells, since they express important regulators of VE as well as crucial regulators of mesoderm formation and gastrulation. These different genetic signatures can account for the different developmental potential of these two stem-cell-based systems.

## STAR★Methods

### Key Resources Table

**Table:** 

REAGENT or RESOURCE	SOURCE	IDENTIFIER
**Antibodies**		

Goat polyclonal anti-AP-2 gamma	R&D Systems	Cat# AF5059; RRID: AB_2255891
Mouse monoclonal anti-AP-2 gamma	Santa Cruz Biotechnology	Cat# sc-12762; RRID: AB_667770
Goat polyclonal anti-Brachyury	R&D Systems	Cat# AF2085; RRID: AB_2200235
Mouse monoclonal anti-Cdx2	BioGenex	Cat# MU392-UC; RRID: AB_2335627
Rabbit monoclonal anti-Cdx2	Abcam	Cat# ab76541; RRID: AB_1523334
Rat monoclonal anti-Cerberus 1	R&D Systems	Cat# MAB1986; RRID: AB_2275974
Goat polyclonal anti-Dkk1	R&D Systems	Cat# AF1096; RRID: AB_354597
Rabbit polyclonal anti-TBR2 / Eomes	Abcam	Cat# ab23345; RRID: AB_778267
Rabbit monoclonal anti-FoxA2 / HNF3	Cell Signaling Technology	Cat# 8186; RRID: AB_10891055
Mouse monoclonal anti-GATA-4	Santa Cruz Biotechnology	Cat# sc-25310; RRID: AB_627667
Goat polyclonal anti-GATA-6	R&D Systems	Cat# AF1700; RRID: AB_2108901
Rat monoclonal anti-GFP	Nacalai Tesque	Cat# GF090R; RRID: AB_2314545
Rabbit polyclonal anti-laminin	Sigma-Aldrich	Cat# L9393; RRID: AB_477163
Goat polyclonal anti-Lefty	R&D Systems	Cat# AF746; RRID: AB_355566
Mouse monoclonal anti-N-Cadherin	BD Biosciences	Cat# 610920; RRID: AB_2077527
Mouse monoclonal anti-Oct-3/4	Santa Cruz Biotechnology	Cat# sc-5279; RRID: AB_628051
Goat polyclonal anti-Otx2	R&D Systems	Cat# AF1979; RRID: AB_2157172
Rat monoclonal anti-Podocalyxin	R&D Systems	Cat# MAB1556; RRID: AB_2166010
Rabbit monoclonal anti-RUNX1 / AML1	Abcam	Cat# ab92336; RRID: AB_2049267
Goat polyclonal anti-Sox17	R&D Systems	Cat# AF1924; RRID: AB_355060
Donkey anti-Mouse IgG (H+L) Highly Cross-Adsorbed Secondary Antibody, Alexa Fluor 488	Thermo Fisher Scientific	Cat# A-21202; RRID: AB_141607
Donkey anti-Rabbit IgG (H+L) Highly Cross-Adsorbed Secondary Antibody, Alexa Fluor 488	Thermo Fisher Scientific	Cat# A-21206; RRID: AB_2535792
Donkey anti-Goat IgG (H+L) Cross-Adsorbed Secondary Antibody, Alexa Fluor 488	Thermo Fisher Scientific	Cat# A-11055; RRID: AB_2534102
Donkey anti-Mouse IgG (H+L) Highly Cross-Adsorbed Secondary Antibody, Alexa Fluor 568	Thermo Fisher Scientific	Cat# A10037; RRID: AB_2534013
Donkey anti-Rabbit IgG (H+L) Highly Cross-Adsorbed Secondary Antibody, Alexa Fluor 568	Thermo Fisher Scientific	Cat# A10042; RRID: AB_2534017
Donkey anti-Goat IgG (H+L) Cross-Adsorbed Secondary Antibody, Alexa Fluor 568	Thermo Fisher Scientific	Cat# A-11057; RRID: AB_2534104
Donkey anti-Mouse IgG (H+L) Highly Cross-Adsorbed Secondary Antibody, Alexa Fluor 647	Thermo Fisher Scientific	Cat# A-31571; RRID: AB_162542
Donkey anti-Rabbit IgG (H+L) Highly Cross-Adsorbed Secondary Antibody, Alexa Fluor 647	Thermo Fisher Scientific	Cat# A-31573; RRID: AB_2536183
Donkey anti-Goat IgG (H+L) Cross-Adsorbed Secondary Antibody, Alexa Fluor 647	Thermo Fisher Scientific	Cat# A-21447; RRID: AB_2535864
Donkey Anti-Rat IgG H&L (Alexa Fluor® 647) preadsorbed antibody	Abcam	Cat# ab150155; RRID: AB_2813835

**Bacterial and Virus Strains**		

5α-competent *E.coli*	New England Biolabs	C2987I

**Chemicals, Peptides, and Recombinant Proteins**		

CHIR99021	Cambridge Stem Cell Institute	N/A
PD0325901	Cambridge Stem Cell Institute	N/A
Leukaemia inhibitory factor	Cambridge Stem Cell Institute	N/A
FGF2	Cambridge Stem Cell Institute	N/A
Recombinant Mouse FGF-4 (aa 67-202)	R&D Systems	Cat# 7486-F4-025
SB431542	STEMCELL Technologies	Cat# 72234
Y-27632	STEMCELL Technologies	Cat# 72304
inDrops v3 barcoding oligonucleotides	Briggs et al., 2018	N/A

**Critical Commercial Assays**		

QuantiTect Reverse Transcription Kit	Qiagen	Cat# 205310
SYBR Green PCR Master Mix	Applied Biosystems	Cat# 4368708
Gateway BP Clonase II Enzyme mix	Invitrogen	Cat# 11789-100
Gateway LR Clonase II Enzyme mix	Invitrogen	Cat# 11791-100
Lipofectamine 3000 Transfection Reagent	Invitrogen	Cat# L3000001

**Deposited Data**		

E4.5 blastocysts single-cell RNA sequencing data	Sozen et al., 2019	GSE134240
E5.5, E6.5 embryo; day 4 ETX and iETX-embryos	This paper	GSE161947
Code for analysis of single-cell RNA sequencing data	This paper	https://github.com/fhlab/scRNAseq_inducedETX

**Experimental Models: Cell Lines**		

Mouse: CAG-GFP/tetO-mCherry/tetO-Gata4 ESCs	This paper	N/A
Mouse: Cerl-GFP mouse ESCs	Mesnard et al., 2004	N/A
Mouse: Cerl-GFP/tetO-Gata4 ESCs	This paper	N/A
Mouse: Bry-GFP ESCs	Lacaud et al., 2004	N/A
Mouse: Snail-YFP ESCs	Dr. Robert Weinberg (Whitehead Institute for Biomedical Research, USA) Dr. Michaela Frye (Stem Cell Institute, University of Cambridge, UK)	N/A
Mouse: Confetti TS cells	Prof. Jenny Nichols (Stem Cell Institute, University of Cambridge, UK)	N/A
Mouse: Wildtype TS cells	Sozen et al., 2019	N/A

**Experimental Models: Organisms/Strains**		

Mouse: CD-1	Charles River	N/A
Mouse: F1	Charles River	N/A

**Oligonucleotides**		

PCR primer: Gata4-AttB Forward: GGGGACAAGTTTGTACAAAAA AGCAGGCT	This paper	N/A
PCR primer: Gata4-AttB Reverse: GGGGACCACTTTGTACAAGA AAGCTGGGT	This paper	N/A
PCR Primer: M13 Forward: GTAAAACGACGGCCAG	Gateway Cloning Manual	tools.thermofisher.com
PCR Primer: M13 Reverse: CAGGAAACAGCTATGAC	Gateway Cloning Manual	tools.thermofisher.com
qPCR primer: *Gapdh* Forward: CGTATTGGGCGCCTGGTCAC	This paper	N/A
qPCR primer: *Gapdh* Reverse: ATGATGACCCTTTTGGCTCC	This paper	N/A
qPCR primer: *Gata4* Forward: ATGGGCACAGCAGCTCCATGTC	Boroviak et al., 2014	N/A
qPCR primer: *Gata4* Reverse: TGCATAGCCTTGTGGGGACAGC	Boroviak et al., 2014	N/A
P7 reverse PCR primer :CAAGCAGAAGACGGCAT ACGAGATGGGTGTCGGGTGCAG	Briggs et al., 2018	N/A
P5 primer ETX4_rep1:AATGATA CGGCGACCACCGAGATCTA CACAACTTGACTCGTCGGCAGCGTC	This study	N/A
P5 primer ETX4_rep2:AATGATA CGGCGACCACCGAGATCTACA CCCTATGCCTCGTCGGCAGCGTC	This study	N/A
P5 primer ETX4_rep3:AATGAT ACGGCGACCACCGAGATCTAC ACTCTGCAAGTCGTCGGCAGCGTC	This study	N/A
P5 primer ETX4_rep3:AATGAT ACGGCGACCACCGAGATCTACA CTCTGCAAGTCGTCGGCAGCGTC	This study	N/A
P5 primer iETX4:AATGATACG GCGACCACCGAGATCTACACT TGAATAGTCGTCGGCAGCGTC	This study	N/A
P5 primer E5.5:AATGATA CGGCGACCACCGAGATCTACAC CGTTACCATCGTCGGCAGCGTC	This study	N/A
P5 primer E6.5_rep1:AATGAT ACGGCGACCACCGAGATCTACAC CCTATGCCTCGTCGGCAGCGTC	This study	N/A
P5 primer E6.5_rep2:AATGATAC GGCGACCACCGAGATCTAC ACTCTGCAAGTCGTCGGCAGCGTC	This study	N/A

**Recombinant DNA**		

pSAM2-mCherry-Gata4	Lalit et al., 2016; Timothy Kamp (Stem Cell and Regenerative Medicine Centre, University of Wisconsin – Madison, USA)	Addgene plasmid # 72690
PB-tetO-hygro	Dr. José Silva (Stem Cell Institute, University of Cambridge, UK)	N/A
pBAse	Dr. José Silva (Stem Cell Institute, University of Cambridge, UK)	N/A
rtTA-zeocyin	Dr. José Silva (Stem Cell Institute, University of Cambridge, UK)	N/A

**Software and Algorithms**		

Imaris	Oxford Instruments	https://imaris.oxinst.com/
Fiji	Schindelin et al., 2012	https://imagej.net/Fiji
NDSAFIR 3.0	Boulanger et al., 2010	https://gitlab.inria.fr/serpico
Smart Denoise	Gurdon Institute	N/A
StackReg (ImageJ/Fiji plugin)	Thévenaz et al., 1998	http://bigwww.epfl.ch/thevenaz/stackreg/
MultiStackReg (ImageJ/Fiji plugin)	Brad Busse (Division of Program Coordination, Planning and Strategic Initiatives, NIH - USA)	https://github.com/miura/MultiStackRegistration
Template Matching and Slice Alignment (ImageJ/Fiji plugin)	Qingzong Tseng (Aix-Marseille Université, France	https://sites.google.com/site/qingzongtseng/template-matching-ij-plugin
Chemotaxis and Migration Tool	ibidi	https://ibidi.com/chemotaxis-analysis/171-chemotaxis-and-migration-tool.html
Prism 8	GraphPad	https://www.graphpad.com/scientific-software/prism/
Bcl2fastq	Illumina	N/A
FastQC tool	Andrews, 2010	https://www.bioinformatics.babraham.ac.uk/projects/fastqc/
Pheniqs	Biosails	https://github.com/biosails/pheniqs
zUMIs	Parekh et al., 2018	https://github.com/sdparekh/zUMIs
Scanpy	Wolf et al., 2018	https://github.com/theislab/scanpy
Scrublet	Wolock et al., 2019	https://github.com/AllonKleinLab/scrublet
SciPy	Virtanen et al., 2020	N/A
Seurat v3	Stuart et al., 2019	https://github.com/satijalab/seurat
DAVID Gene Ontology	Huang et al., 2009b, 2009a	https://david.ncifcrf.gov/

**Other**		

AggreWell400	STEMCELL Technologies	Cat# 34415
Anti-Adherence Rinsing Solution	STEMCELL Technologies	Cat# 07010
Gri3D PEG-hydrogel dishes	SunBioscience	https://sunbioscience.ch/products/
Leica SP5	Leica Microsystems	N/A
Leica SP8	Leica Microsystems	N/A
Zeiss Axiovert 200M	Zeiss	N/A

### Resource availability

#### Lead Contact

Further information and requests for resources and reagents should be directed to and will be fulfilled by the Lead Contact, Magdalena Zernicka-Goetz (mz205@cam.ac.uk).

#### Materials Availability

All unique/stable reagents generated in this study are available from and will be provided by the Lead Contact with a completed Materials Transfer Agreement.

#### Data and Code Availability

The accession number for the single-cell sequencing data reported in this paper is Gene Expression Omnibus: GSE134240 (https://www.ncbi.nlm.nih.gov/geo/query/acc.cgi?acc=GSE161947).The code used in these analyses is available at https://github.com/fhlab/scRNAseq_inducedETX.

### Experimental Model and Subject Details

#### Cell Lines and Culture Conditions

Cell lines used in this study include:

- CAG-GFP/tetO-mCherry mouse ESCs (constitutive GFP expression in the membrane; transient mCherry expression upon Dox treatment). The parent CAG-GFP/tetO-mCherry ESC line was derived from an existing mouse line with constitutive CAG-GFP expression and Dox-induced transient mCherry expression. This line was generated by breeding CAG-GFP (Rhee et al., 2006) reporter mice and tetO-mCherry Histone mice (Egli et al., 2007). For the purpose of this study, an independent Dox-inducible Gata4-expressing cassette was introduced into the CAG-GFP/tetO-mCherry ES lines by piggyBac-based transposition, as described below, thus mCherry and Gata4 are regulated by two, independent Dox-responsive promoters.
- CAG-GFP/tetO-mCherry/tetO-Gata4 ESCs generated in-house.
- Cerl-GFP mouse ESCs (GFP expression under the control of the Cerl-promoter) were derived from a published Cerl-GFP mouse line (Mesnard et al., 2004).
- Cerl-GFP/tetO-Gata4 ESCs generated in-house.
- Bry-GFP ESCs (Lacaud et al., 2004).
- Snail-YFP ESCs were derived from Snail-YFP transgenic mice (Ye et al., 2015), which were a generous gift of Dr. Robert Weinberg (Whitehead Institute for Biomedical Research USA) and Dr. Michaela Frye (University of Cambridge, UK).
- Mouse Confetti TS cells were a generous gift of Prof. Jenny Nichols (Stem Cell Institute, Cambridge, UK). Because they were not treated with Tamoxifen, they did not express any reporter.
- Wildtype TS cells were generated in house from CD1 mice (Sozen et al., 2019).

The sex of the cell lines is not known because we did not genotype them to determine it.

All cell lines were routinely tested every two weeks to ensure that they were not contaminated with mycoplasma.

Mouse embryonic stem cells were cultured on gelatinised plates at 37°C, 5% CO2, 21% O2 in N2B27 which is comprised of 50% Neurobasal-A (Gibco 10888022), 50% DMEM/F-12 (Gibco 21331020), 0.5% N2 (in-house), 1% B27 (Gibco 10889038), 2mM GlutaMAX (Gibco 35050038), 0.1mM 2-mercaptoethanol (Gibco 31350010) and 1% penicillin/streptomycin (Gibco 15140122). N2B27 was supplemented with 3μM CHIR99021 (Cambridge Stem Cell Institute), 1μM PD0325901 (Cambridge Stem Cell Institute) and 10 ng ml^-1^ leukaemia inhibitory factor (Cambridge Stem Cell Institute). Mouse trophoblast stem (TS) cells were cultured on mitotically inactivated mouse embryonic fibroblasts (MEFs, Insight Biotechnology, ASF-1201) in feeder cell (FC) medium which contained Dulbecco’s modified essential medium (Gibco 41966052), 15% foetal bovine serum (Cambridge Stem Cell Institute), 1mM sodium pyruvate (Gibco 11360039), 2mM GlutaMAX (Gibco 35050038), 1% MEM non-essential amino acids (Gibco 11140035), 0.1mM 2-mercaptoethanol (Gibco 31350010) and 1% penicillin/streptomycin (Gibco 15140122). FC medium was supplemented with 1 μg ml^-1^ heparin (Sigma-Aldrich H3149-25KU), 25 ng ml^-1^ FGF2 (Cambridge Stem Cell Institute) and 25 ng ml^-1^ FGF4 (R&D Systems 7486-F4-025) (FC F42H). Passaging of ES and TS cells was performed when they were at 70% confluency as follows: cells were washed once in 1x PBS (Life Technologies 10010056) and trypsinised (Trypsin-EDTA 0.05% Life Technologies 25300054) for 4 minutes at 37°C. The reaction was stopped by adding 2 mL of FC, cells were dissociated by pipetting gently 4-5 times and centrifuged for 4 minutes at 200 x g. TS cells were then resuspended in FC F42H culture media and plated onto MEF-coated plates in 1:20 dilution. ESCs were washed once with 1 mL of 1x PBS, centrifuged again, resuspended in N2B27 2iLIF and plated at 1:10 or 1:20 onto gelatine-coated plates.

#### Mouse Model

Mice were handled following national and international guidelines. All experiments performed were under the regulation of the Animals (Scientific Procedures) Act 1986 Amendment Regulations 2012 and were reviewed by the University of Cambridge Animal Welfare and Ethical Review Body (AWERB). Experiments were approved by the Home Office. Animals were inspected daily and those showing signs of any health concern or condition were promptly culled by cervical dislocation. All experimental mice were free of pathogens and were on a 12-12 hour light-dark cycle, with unlimited access to water and food. Temperature in the facility was controlled and maintained at 21°C. Mice for post-implantation embryo recovery (CD-1 females and males from Charles River, acclimatised for 1 week prior to use) were utilised from 6 weeks of age. Female and males were naturally mated and kept together for up to five days or until a plug was found; females were inspected daily for plugs. Females were culled by cervical dislocation 5.5, 5.75, 6.5, 6.75 or 7.5 days after a plug was found. Embryos were dissected out of the deciduae in M2 medium (Sigma M7167). For chimera experiments, F1 females (Charles River, 1 week of acclimatisation prior to use) at 5-6 weeks of age were super-ovulated by injection of 7.5 IU of pregnant mares’ serum gonadotropin (Intervet) and 7.5 IU of human chorionic gonadotropin (Intervet) after 48 hours and were mated with F1 males (Charles River, 1 week of acclimatisation prior to use). Pregnant F1 females were culled at E2.5 by cervical dislocation to recover embryos by uterine and oviduct flushing in M2 medium.

### Method details

#### Formation of ES Cell Aggregates and iETX embryos

To prepare the AggreWell plate (STEMCELL Technologies 34415), 500μl of anti-adherence rinsing solution (STEMCELL Technologies 07010) was added to each well. The plate was then centrifuged at 2,000 x g for 5 minutes and was incubated for 20 minutes at room temperature. Rinsing solution was then aspirated from the well and 1ml of PBS was added to wash each well. 500μl of FC medium was added to each well after aspirating the PBS.

To prepare ESCs for generating ES aggregates, Doxycycline (1 μg/mL) (Sigma-Aldrich D9891-5G) was added to CAG-GFP tetO-Gata4 ESCs 6 hours prior to plating in AggreWell. ESCs were washed once with 1x PBS, and trypsinised with 0.05% trypsin-EDTA (ThermoFisher Scientific) for 4 minutes at 37°C. The reaction was stopped by adding 2 mL of FC. Cells were dissociated gently by pipetting for 4-5 times and centrifuged at 200 x g for 4 minutes. The cell pellet was washed once with 1x PBS, centrifuged again and resuspended in 1-2 mL of FC. Cell suspensions containing 1) 12,000 Doxycycline-treated CAG-GFP tetO-Gata4 ESCs, or 2) 12,000 untreated CAG-GFP tetO-Gata4 ESCs, or 3) a mixture of 6,000 Doxycycline-treated and 6,000 untreated CAG-GFP tetO-Gata4 ESCs were pelleted again by centrifugation. After resuspending in 1ml of FC medium, the cell suspension was added dropwise to the AggreWell and the plate was centrifuged at 100 x g for 3 minutes. 1ml of fresh FC medium was added each day after removing 1ml of medium from the well. ES aggregates were collected and fixed after 1 day or 3 days.

To generate iETX embryos, Doxycycline was added to CAG-GFP tetO-Gata4 ESCs for 6 hours. TS cells were trypsinised and were added to a gelatinised plate to deplete the MEFs for 20 minutes at 37°C. CAG-GFP WT ESCs and CAG-GFP tetO-Gata4 ESCs were subsequently trypsinised. Cell suspensions with 19,200 TS cells, 6,000 CAG-GFP WT ESCs and 6,000 CAG-GFP tetO-Gata4 ESCs were mixed and pelleted by centrifugation. The cell pellet was resuspended in 1ml of FC medium with 7.5 nM ROCK inhibitor (Y27632, STEMCELL Technologies 72304). After adding the cell mixture dropwise to the AggreWell, the plate was centrifuged at 100 x g for 3 minutes. On the next day, media change was performed twice by removing 1ml of medium from each well and adding 1ml of fresh FC medium without ROCK inhibitor. On day 2, media change was performed once to replace 1ml of medium with 1ml of fresh FC medium. On day 3, 1.2 ml of medium was removed from each well and 1.5ml of IVC1 was added, after equilibrating for 20 minutes in the incubator. IVC1 (Bedzhov et al., 2014) is made of advanced DMEM/F12 (Gibco, 21331-020) supplemented with 20% (v/v) FBS, 2 mM GlutaMax, 1% v/v penicillin–streptomycin, 1X ITS-X Thermo Fisher Scientific, 51500-056), 8 nM β-estradiol, 200 ng/ml progesterone and 25 μM N-acetyl-L-cysteine. On day 4, iETX embryos in the AggreWell were transferred to CELLSTAR 6 well multiwell plate for suspension culture (Greiner Bio-One 657185) with 5ml of IVC1 (with FBS at 30% v/v) per well. On day 5 IVC1 was replaced with fresh IVC2 (30% Knockout Serum Replacement instead of FBS, Thermo Fisher 10828010).

#### Chimera

To generate chimeras using CAG-GFP tetO-Gata4 ESCs and mouse embryos, Doxycycline was first added to CAG-GFP tetO-Gata4 ESCs 6 hours prior to the experiment. Mouse embryos at E2.5 before compaction were recovered from F1 females that were super-ovulated by injection of 7.5 IU of pregnant mares’ serum gonadotropin (Intervet) and 7.5 IU of human chorionic gonadotropin (Intervet) after 48 hours and were mated with F1 males. Embryos were recovered in M2 medium by flushing the oviducts. After transferring to KSOM (Millipore MR-020P-5F), the embryos were cultured in the incubator at 37°C in 5% CO_2_. Meanwhile, CAG-GFP tetO-Gata4 ESCs were dissociated by 2 minutes of trypsinisation at 37°C and the resulting cell clumps were aggregated with the recovered embryos in KSOM. The chimeras were cultured for 48 hours until E4.5.

#### Nodal Inhibitory Treatment

iETX embryos were collected at day 4 and incubated in SB431542 (STEMCELL Technologies 72234) for 24 hours at a concentration of 10 μM (Kyprianou et al., 2020). Following Nodal inhibitor treatment, they were fixed and processed for immunofluorescence (below).

#### Live Imaging

Live imaging was performed using an SP8 scanning confocal microscope with a 25X objective. iETX embryos were imaged on a glass-bottom dish and were kept in a humidified chamber with 5.6% CO_2_ and 21% O_2_ during the imaging. Images were captured every 20 minutes (AVE migration and gastrulation movies) with a z-step of 2 μm. For self-organization movies, cells were imaged after seeding on Gri3D PEG-hydrogel dishes provided by SunBioscience (Geneva, Switzerland), set up according to the manufacturer’s guidelines and samples were imaged every 60 minutes. Samples were imaged on a Zeiss Axiovert 200M connected to a 3i CSU-W Spinning Disk Confocal system with an OBIS 488nm and an OBIS 561-nm LS laser and exported using Slidebook.

#### Plasmids and Transfection

Gata4 cDNA was PCR-amplified from pSAM2-mCherry-Gata4 using the Gata4/AttB primers (see Key Resources Table). The primers were designed as outlined in the Gateway cloning manual. Because the plasmid already contained attB sites, there was no need to incorporate parts of the Gata4 open reading frame in the primer design. pSAM2-mCherry-Gata4 was a gift from Timothy Kamp (Stem Cell and Regenerative Medicine Centre, University of Wisconsin – Madison, USA; Addgene plasmid # 72690; http://n2t.net/addgene:72690; RRID:Addgene_72690, (Lalit et al., 2016)). It was subsequently cloned into PB-tetO-hygromycin by Gateway technology (Thermo Fisher Scientific), according to the manufacturer’s instructions. Clones were verified by sequencing. Transformations were performed using 5α-competent *E.coli* following the manufacturer’s guidelines (New England Biolabs C2987I). To generate ESCs with Doxycycline inducible Gata4, PB-tetO-hygro-Gata4, pBAse and rtTA-zeocyin (0.25 μg/each/reaction) were transfected into 12,000 CAG-GFP ESCs using Lipofectamine 3000 Transfection Reagent (Invitrogen L3000001), followed by antibiotics selection for 7 days with hygromycin (1:250; Gibco 10687010) and zeocyin (1:1000; InvivoGen ant-zn-1). The PB-tetO-hygro, pBAse and rtTA-zeocyin were generously gifted by Dr. José Silva (Stem Cell Institute, Cambridge, UK).

#### RNA Extraction and qRT-PCR

Total RNA from cell pellet was extracted using Trizol Reagent (Invitrogen 15596-026) and reverse transcribed into cDNA using QuantiTect Reverse Transcription Kit (Qiagen 205310) according to the manufacturer’s instructions. qRT-PCR was performed using SYBR Green PCR Master Mix (Applied Biosystems 4368708) and StepOnePlus Real-Time PCR System (Applied Biosystems). Fold change in *Gata4* mRNA expression was determined by ΔΔCt method using *Gapdh* as endogenous control. See Table for *Gapdh* and *Gata4* (Boroviak et al., 2014) primer sequences.

#### Immunofluorescence

iETX embryos and natural mouse embryos were fixed with 4% paraformaldehyde at room temperature for 20 minutes and washed with PBST (PBS with 0.1% Tween 20) for three times for 5 minutes each. Samples were then permeabilised in permeabilization buffer (0.1 M glycine and 0.3% Triton X-100 in PBS) for 30 minutes at room temperature, followed by three washes with PBST for 5 minutes. Samples were incubated with primary antibodies diluted in blocking buffer (10% FBS and 0.1% Tween 20 in PBS) at 4°C overnight. After washing with PBST for three times for 5 minutes, samples were incubated with secondary antibodies at 4°C overnight or for 2 hours at room temperature followed by another three washes with PBST for 5 minutes before imaging.

#### scRNA-seq Sample Preparation and Dissociation

After recovery, natural embryos and iETX embryos were cut to pieces, transferred to a Falcon tube, centrifuged, washed in PBS and incubated in Tryple Express (Gibco 12604013) for 15 minutes at 37°C, with vigorous pipetting every 5 minutes to dissociate to single cells. If there were clumps left, the incubation was extended for an additional 5 minutes at 37°C and the sample was pipetted further. Samples were filtered to remove large clumps, centrifuged at 200 x g for 5 minutes and resuspended in PBST (PBS with 0.02% Tween20) and then processed for incapsulation (see below). For E5.5 and E6.5, 1 full litter was dissociated (12 embryos). For ETX embryos and iETX embryos, 15 samples each were dissociated. Single cell sequencing data from 20 E4.5 blastocysts was obtained from (Sozen et al., 2019, GEO: GSE134240).

#### scRNA-seq Library Preparation and Sequencing

Libraries were prepared according to the inDrops workflow (Klein et al., 2015; Zilionis et al., 2017) with v3 barcoding scheme (Briggs et al., 2018). Briefly, polyacrylamide beads were generated and barcoded to obtain a diversity of 147,456 barcodes. Single-cell suspensions were diluted to a concentration of 120,000 cells per ml and co-encapsulated with the barcoded beads and reverse transcriptase and lysis mix. Fractions of ∼2,900 cells were collected in 1.5 ml Eppendorf tubes pre-filled with 200 μl of mineral oil and incubated at 50°C for 2 hours and 70°C for 20 minutes. The droplets were then de-emulsified and further amplified using second-strand synthesis and *in vitro* transcription. The libraries were then fragmented and reverse transcribed. The final libraries were amplified using limited-cycle PCR and quantified using a Qubit High sensitivity and Bioanalyzer High sensitivity DNA kits. Libraries were pooled at equi-molar ratios and purified using a 1.5x volumetric ratio of AmpureXP beads. The libraries were sequenced on a Nextseq 75 cycle 400M read High Output kit with 5% PhiX spike-in as an internal control. The read cycle distribution was the following: Read1: 61 cycles; Index1: 8 cycles; Index2: 8 cycles; Read2: 14 cycles.

### Quantification and statistical analysis

#### Inclusion Criteria of iETX embryos

All iETX embryos were collected from AggreWell for analysis at 3, 4 or 5 days of development and analysed under a stereo microscope. In all instances, we selected iETX embryos with cylindrical morphology, an epithelialized ES compartment with a lumen and two clearly defined cellular compartments surrounded by an outer cell layer. The TS compartment is more variable in appearance and therefore, even though one would also want an epithelial-looking TS compartment similar to the extra-embryonic ectoderm of natural embryos, we select a wider range of appearances for the TS compartment. After this initial selection, structures containing the appropriate fluorophores were quickly checked under a microscope to ensure the presence of an epithelialized CAG-GFP-positive ES compartment. iETX embryos with the correct body plan of ES and TS compartments surrounded by a visceral endoderm-like layer are then transferred to equilibrated media to continue their culture. When selecting at day 5, however, we expect the lumen of the ES and TS compartment to be merged. We provide visual examples of what we consider good iETX embryos at day 4 in Figure 2B.

#### iETX embryo Cell Lineage Quantification

Cell lineage quantifications were performed using the “Spot” function in Imaris (Bitplane). For the VE-like layer, the spots were set at a diameter of 9 μm. For ESCs and TS cells the spots were set at a diameter of 6 μm. Following automatic detection, the spots were manually curated to remove erroneous cell calls and to include cells that were missed. Because of the loss of resolution past the midpoint of the iETX embryos, cells were only quantified up to the midpoint of each iETX embryo and then the number of cells obtained was multiplied by 2 to obtain the total number of cells.

#### Image Acquisition, Processing and Analysis

Images were acquired using Leica SP5 and SP8 confocal microscopes (Leica Microsystems) with 40× oil objective and 25× water objective, respectively. A 405 nm diode laser (DAPI), a 488 nm argon laser (Alexa Fluor 488), a 543 nm HeNe laser (Alexa Fluor 568) and a 633 nm HeNe laser (Alexa Fluor 647) were used to excite the fluorophores. Images were taken with a z-step of 1.2-5μm. Fiji (Schindelin et al., 2012) and NDSAFIR 3.0 (Boulanger et al., 2010), Photoshop and the Smart Denoise (Gurdon Institute) were used to process and analyse the images. In Figure 4A, the Bry antibody in the natural embryo shown had strong non-specific membrane signal, which has been reported elsewhere (Morgani et al., 2018). To reduce the background signal, we used the Oct4 signal in that image to create a mask of the epiblast and subtract the Bry background from the nuclear signal. This is the only case in which we have edited an image this way.

#### Tracking of AVE Migration

Images from time lapse video were processed with Fiji. To correct for the drifting of iETX-embryo during imaging, images were first aligned using StackReg plugin (Thévenaz et al., 1998), MultiStackReg (Brad Busse, Division of Program Coordination, Planning and Strategic Initiatives, NIH - USA) and Template Matching and Slice Alignment plugin (Qingzong Tseng, Aix-Marseille Université, France). The movements of individual Cerl-GFP-positive cells were then tracked with Manual Tracking plugin which generated the migration trajectories. The plugin also produced a table of the coordinates of the tracked cells in each time frame which was later imported to Chemotaxis and Migration Tool (iBidi) to calculate the migration directionality and migration speed. The polar histogram of migration directionality was generated using R.

#### Quantification of Cerl-GFP Fluorescence

In Fiji, a rectangle encompassing the length of the ES compartment and wide enough to contain all the Cerl-GFP positive cells was drawn over a day 5 iETX embryo. Using the plot function, signal intensity as a function of distance was obtained and exported. The same area was sequentially used to export Cerl-GFP, Eomes, Phalloidin and DAPI intensities for each iETX embryo. If a single Cerl-GFP peak was observed on one side of the iETX embryo, the Cerl-GFP signal was considered asymmetric, otherwise it was considered symmetric. To normalize the length of the structure, each point of the length was divided by the total length of the ES compartment. To normalize the fluorescence, each fluorescence value was divided by the highest fluorescence value. Normalised samples were combined (asymmetric with asymmetric and symmetric with symmetric) and displayed as a Lowess curve generated in Prism.

#### scRNA-seq Bioinformatic Analysis

The BCL files were converted to Fastq files using Illumina’s bcl2fastq software. The sequenced libraries were quality-inspected using the FastQC tool (Andrews, 2010) and de-multiplexed using the Pheniqs tool from biosails. The fastq files were further filtered, mapped to a mouse GRCm38 reference genome with GRCm38.99 gtf annotation and deduplicated using the zUMIs pipeline (Parekh et al., 2018). The count matrices were then imported in Scanpy (Wolf et al., 2018) using the scanpy.read() function. The Scrublet module (Wolock et al., 2019) was used to predict doublet scores. Cells with predicted doublet scores lower than 0.2 and with gene counts higher than 1,200 were then selected. The filtered matrices were then concatenated after being converted to a sparse format using the csr_matrix() function from SciPy (Virtanen et al., 2020). Cells were further filtered on ribosomal RNA (percentage of reads mapping to ribosomal RNA between 2.5% and 10%) and mitochondrial RNA content (percentage of reads mapping to mitochondrial RNA between 2% and 12%). The matrix was then normalized, regressed out for number of UMI counts, ribosomal RNA and mitochondrial RNA content using the scanpy.pp.regress_out() function, scaled and a UMAP dimensional reduction was computed. To plot the correlation between ETX, iETX, E4.5, E5.5 and E6.5 and to match the sample size and number of cells per lineage of E4.5 (E4.5 dataset was from (Sozen et al., 2019)), each sample was downsampled to randomly contain 41 cells from the endoderm lineage, 33 cells from the embryo lineage and 22 cells from the trophectoderm lineage. Correlation matrices were computed with scanpy.pl.correlation_matrix function using Pearson correlation. For cell type annotation, a combination of leiden clustering (obtained using scanpy.tl.leiden) and a marker-based approach based on Pijuan-Sala et al.’s (Pijuan-Sala et al., 2019) annotations was utilised with the following normalized marker expression levels: 1) parietal endoderm annotation for iETX cells of the endoderm compartment with Lamb1 expression higher than 3.5, 2) primitive streak annotation for cells of the embryo compartment with Nanog and Eomes expression level higher than 1.5, 3) nascent mesoderm annotation for cells of the embryo compartment with Mesp1 expression level higher than 1.5, 4) Visceral endoderm cells from iETX embryos with Otx2 expression level higher than 1.5, 5) ExE and differentiating ExE annotations were given according to the resulting leiden clustering and also using Cdx2 for the trophoblast stem cells/extraembryonic ectoderm cells and Gata2 for the differentiated trophoblast cell types (see Figure S7A).

To perform pairwise analysis of differentially expressed genes, the matrices and annotations were loaded into Seurat v3 (Stuart et al., 2019) and pairwise marker comparisons were obtained with a Wilcoxon Rank Sum test using the FindMarkers function. Differentially expressed genes were then plotted in Prism GraphPad to generate volcano plots. Single-cell sequencing data are available on GEO (Gene Expression Omnibus) under accession GSE161947 (https://www.ncbi.nlm.nih.gov/geo/query/acc.cgi?acc=GSE161947) and the code used in these analyses is available at https://github.com/fhlab/scRNAseq_inducedETX.

#### Gene Ontology

Gene Ontology was performed using the online platform DAVID (Huang et al., 2009b, 2009a). Differentially expressed genes from pairwise comparisons were selected by choosing genes with an adjusted p value < 0.05 and enriched in one sample or the other of the pairwise comparison. The list was then uploaded in the DAVID user interface and analysed with the Gene Functional Annotation Clustering tool and the Gene Functional Annotation Table. The first 10 clusters with the highest Enrichment Score (-log p value) were graphed. For the Wnt signalling category identified as enriched in the VE-like layer in comparison with the XEN layer, Wnt-related function of the genes in the list was found using the annotations of the Uniprot database (2019) and provided in Table S2.

#### Statistics

All statistical analyses were performed with GraphPad Prism 8 software. Quantitative data were presented as mean ± SD or SEM as indicated in figure legends or as violin plots with median and quartiles. Prior to statistical significance testing, data were tested for normal distribution with the Shapiro-Wilk test. For normally distributed data, the unpaired or paired Student’s t test was used for 2 groups and One-Way ANOVA with Tukey’s multiple comparison post-hoc test for more than 2 groups. For data that did not follow a normal distribution, Mann-Whitney non-parametric test was used for 2 groups and One-Way ANOVA followed by Kruskal-Wellis non-parametric post-hoc test for more than 2 groups. Wilcoxon matched pairs signed rank test was used for non-parametric paired analysis. A p value < 0.05 was considered significant. Sample size and the number of experimental replicates (n) is indicated in the relevant figure legend. Sample size was not predetermined. For the supplemental figure, the statistical test utilised and the exact p values are in the relevant figure legend. For the main figures, the statistical test utilised and the exact p values are shown below:

Figure 1: A. Unpaired Student’s t test: N2B27 2iLIF: p = 0.0218, FC 2iLIF: p < 0.0001, IDG 2iLIF: p = 0.0020. B. One-way ANOVA, Kruskal-Wallis post-hoc test, p < 0.0001. D. Unpaired Student’s t test, p < 0.0001

Figure 2**: C.** Unpaired Student’s t test p=0.0052. J. One-Way ANOVA; ES compartment, Epiblast (ES/EPI): Tukey post-hoc. ^∗∗∗∗^ p < 0.0001. TS compartment, Extraembryonic ectoderm (TS/ExE): Tukey post-hoc.^∗^ p = 0.0236. XEN layer, VE-like layer and visceral endoderm (XEN/VE): Kruskal-Wallis post-hoc.^∗∗^ p = 0.0077, ^∗∗∗∗^ p < 0.0001.

Figure 3**: E.** Unpaired Student’s t test p = 0.0071. **F.** Unpaired Student’s t test p = 0.0478. **G.** Unpaired Student’s t test p = 0.0079. **H.** Mann-Whitney test p = 0.0003. **I.** Mann-Whitney test p = ns. J. Student’s t test p = 0.0407. L. One-Way ANOVA p < 0.0001, Kruskal-Wallis post-hoc test. ^∗∗∗∗^ p < 0.0001, ^∗∗^ p = 0.0033. **N.** Unpaired Student’s t test p = 0.033. **O.** Unpaired Student’s t test p = ns. **P.** Unpaired Student’s t test p = 0.0194.

Figure 4**: D.** One-way ANOVA, p < 0.0001; Tukey’s multiple comparison post-hoc test: opposite vs Same and Distal, p < 0.0001; Distal vs Same p = ns. F. One-Way ANOVA p<0.0001; Tukey’s multiple comparison post-hoc test: opposite vs Same and Opposite vs Distal p < 0.0001, Same vs Distal p = ns. H. One-Way ANOVA p < 0.0001; Tukey’s multiple comparison post-hoc test: opposite vs Same and Opposite vs Distal p < 0.0001, Same vs Distal p = ns. J. One-Way ANOVA p = 0.0002; Tukey’s multiple comparison post-hoc test: ectopic vs non-ectopic p = 0.0011; ectopic vs. distal p = 0.0007.

Figure 5**: E.** Multiple t tests, p < 0.000001. **H.** Unpaired t test: Bry p < 0.0001, N-Cad p = 0.0049, E-Cad p = 0.0207. **I.** (left) One-Way ANOVA and Tukey multiple comparison, ^∗∗∗∗^ p < 0.0001, ^∗∗^ p = 0.0022. (right) Wilcoxon matched pairs signed rank test, ^∗^ p = 0.0178.

## Supplemental Information

Document S1. Figures S1–S7 and Tables S1 and S2

Document S2. Article plus Supplemental Information
