# Supplementary material for: Inducible Stem-Cell-Derived Embryos Capture Mouse Morphogenetic Events In Vitro
Source: Dev Cell. 2021 Feb 8;56(3):366–382.e9. doi: 10.1016/j.devcel.2020.12.004 (PMC7883308; doi:10.1016/j.devcel.2020.12.004)
Supplement: Document S1. Figures S1–S7 and Tables S1 and S2 [file mmc1.pdf]

Developmental Cell, Volume *56*

Supplemental Information

Inducible Stem-Cell-Derived Embryos

Capture Mouse Morphogenetic Events *In Vitro*

Gianluca Amadei, Kasey Y.C. Lau, Joachim De Jonghe, Carlos W. Gantner, Berna Sozen, Christopher Chan, Meng Zhu, Christos Kyprianou, Florian Hollfelder, and Magdalena Zernicka-Goetz

Figure S1 (related to Figure 1)

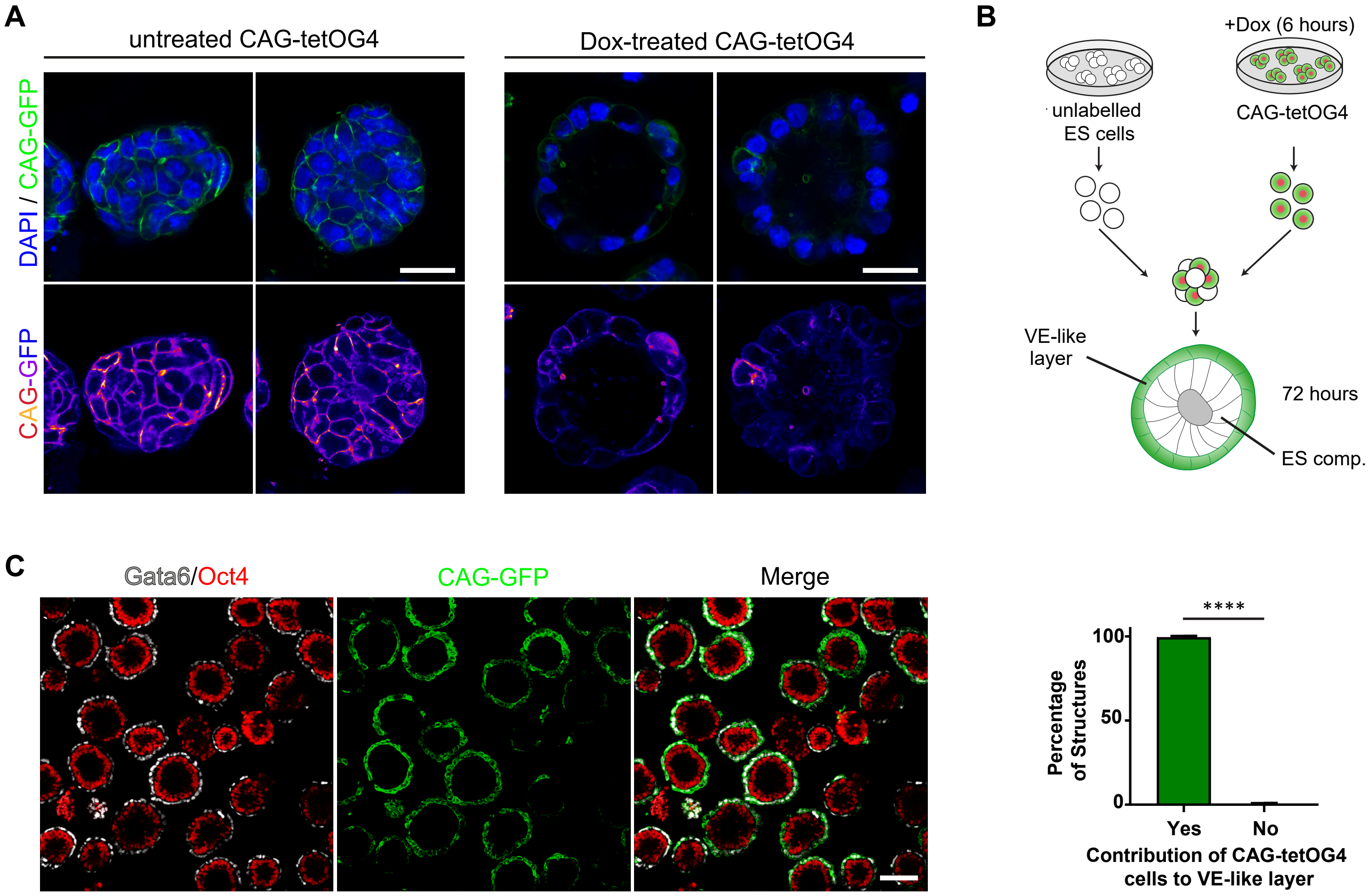

Figure S1. Dox-induced CAG-tetOG4 ES cells downregulate endogenous CAG-GFP and generate the VE-like layer, related to Figure 1. A. Representative cell aggregates generated from either uninduced or Dox-induced CAG-tetOG4 ES cells, collected after 3 days and stained with DAPI (blue) and imaged for endogenous CAG-GFP signal (green in the top row and fire in the bottom row). n=3, scale bar, 30 μm. **B.** Schematic of lineage tracing experiment. Unlabelled ES cells were mixed in 1:1 ratio with Dox-induced CAG-tetOG4 ES cells; aggregates were collected after three days to assess whether the CAG-tetOG4 ES cells generated the VE-like layer or not. **C.** Aggregates generated by combining unlabelled ES cells and Dox-induced CAG-tetOG4 ES cells were collected after 3 days and analysed for Oct4 (red), Gata6 (grey) and CAG-GFP (immunofluorescence with an α-GFP antibody); scale bar,100 μm. Aggregates were scored for contribution of CAG-tetOG4 ES cells to the VE-like layer. If an aggregate had a CAG-GFP+ve VE-like layer, CAG-tetOG4 had contributed to it and the aggregate was scored as “Yes”; if not, it was scored as “No”. Error bar=SD. Yes: 1791/1798, n=3. Paired Student t-test, $p < 0.0001$.

Figure S2 (related to Figure 2)

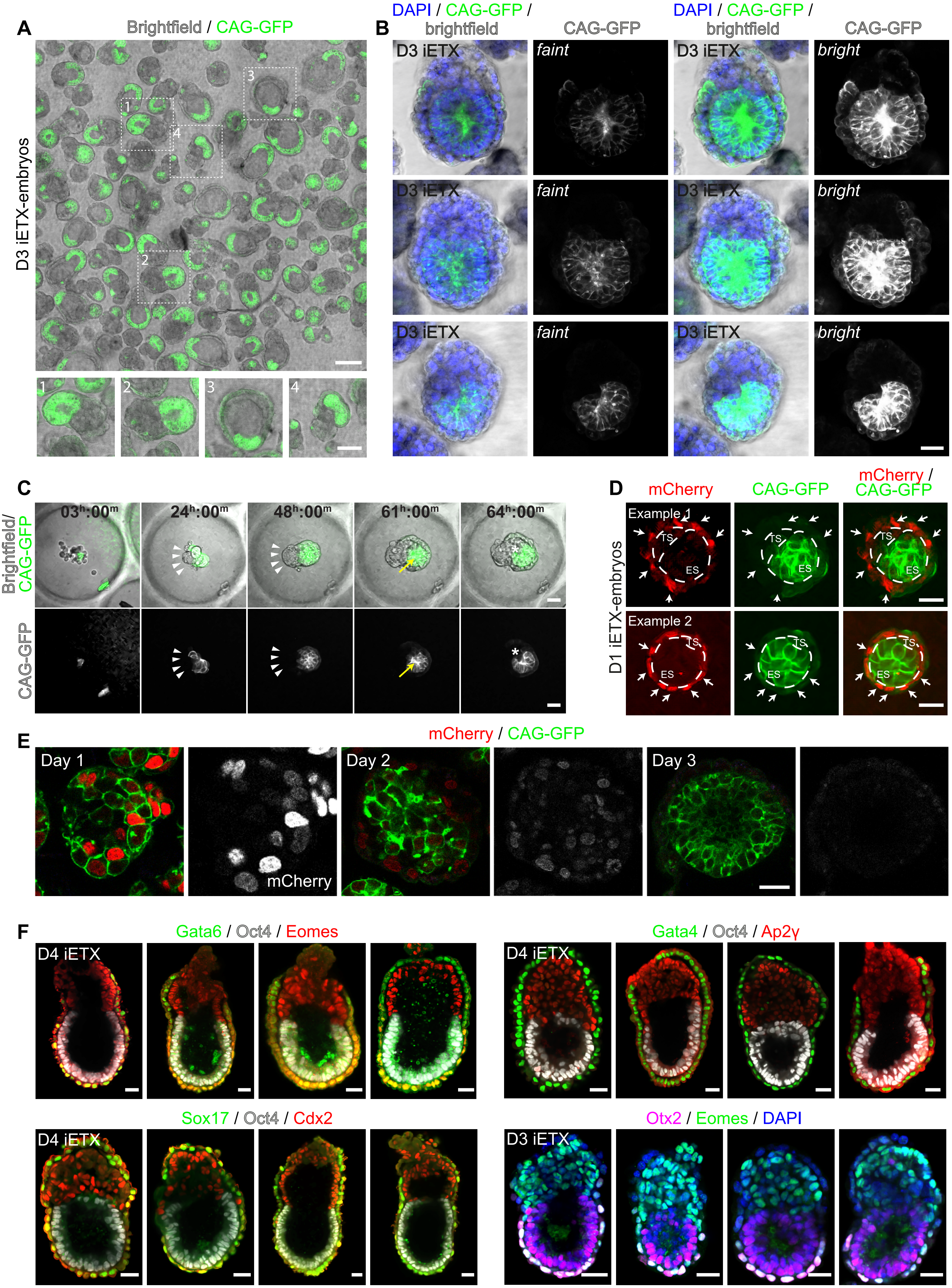

Figure S2. iETX embryos are morphologically similar to mouse post-implantation embryos and express canonical post-implantation markers, related to Figure 2. A. All the structures in a single AggreWell were collected at day 3 of development and imaged to quantify the formation efficiency of iETX embryos. CAG-GFP in green marks the contribution of ES cells and induced CAG-tetOG4 ES cells, but the CAG-GFP signal is downregulated in the VE-like layer (see below). GFP-ve cells are the TS cells. Scale bar, 150 µm. Dashed squares are below to present examples from a typical experiment such as well-formed iETX embryos (1,2), an inflating iETX embryo (3), and a misshapen structure (4). Scale bar, 100 µm. **B.** Representative iETX embryos at day 3 of development stained with DAPI (blue) and analysed for the endogenous CAG-GFP signal in the ES compartment and VE-like layer. At low laser, only the endogenous CAG-GFP signal in the ES compartment is visible, but with high laser, also the CAG-GFP signal in the VE-like layer becomes visible. This indicates downregulation of the CAG-GFP signal in the VE-like layer, and it is similar to what occurs in natural mouse embryos of the same background, as reported (Bedzhov and Zernicka-Goetz, 2014). Scale bar, 30 µm, n=3. **C.** Still images of a time-lapse of iETX embryo formation in an optical PEG hydrogel dish over the course of 64 hours. CAG-GFP wt ES cells and Dox-treated CAG-tetOG4 ES cells are in green, TS cells are not labelled. ES and TS cells are either shown together (top row), or ES cells (Dox-treated and wt) are shown alone (bottom row). White arrowheads highlight TS cells, yellow arrow and asterisk show the forming ES lumen. 6 examples. Scale bar, 30 µm. **D.** 2 examples of iETX embryos at 1 day of development. ES and TS compartment are enclosed with a dashed line. Induced CAG-tetOG4 ES cells transiently express mCherry and are indicated by arrows. 91/109 iETX embryos from 2 independent experiments. Scale bar, 30 µm. **E.** iETX embryo analysed at day 1, day 2 and day 3 of development to assess endogenous CAG-GFP (green) and endogenous mCherry expression (red or grey) after Dox-induction. Scale bar, 100 µm, n=3. **F.** Multiple examples of iETX embryo at day 4 (top rows and bottom left row) and day 3 (bottom right row) of development stained with the indicated combinations of lineage markers: Gata6 (green), Oct4 (grey) and Eomes (red); (41/42); Sox17 (green), Oct4 (grey) and Cdx2 (red); (21/21); Gata4 (green), Oct4 (grey) and Ap2γ (red); (18/19); Otx2 (purple), Eomes (green); DAPI (blue) (27/31); n=3 each. All scale bars, 30 µm.

Figure S3 (related to Figure 3)

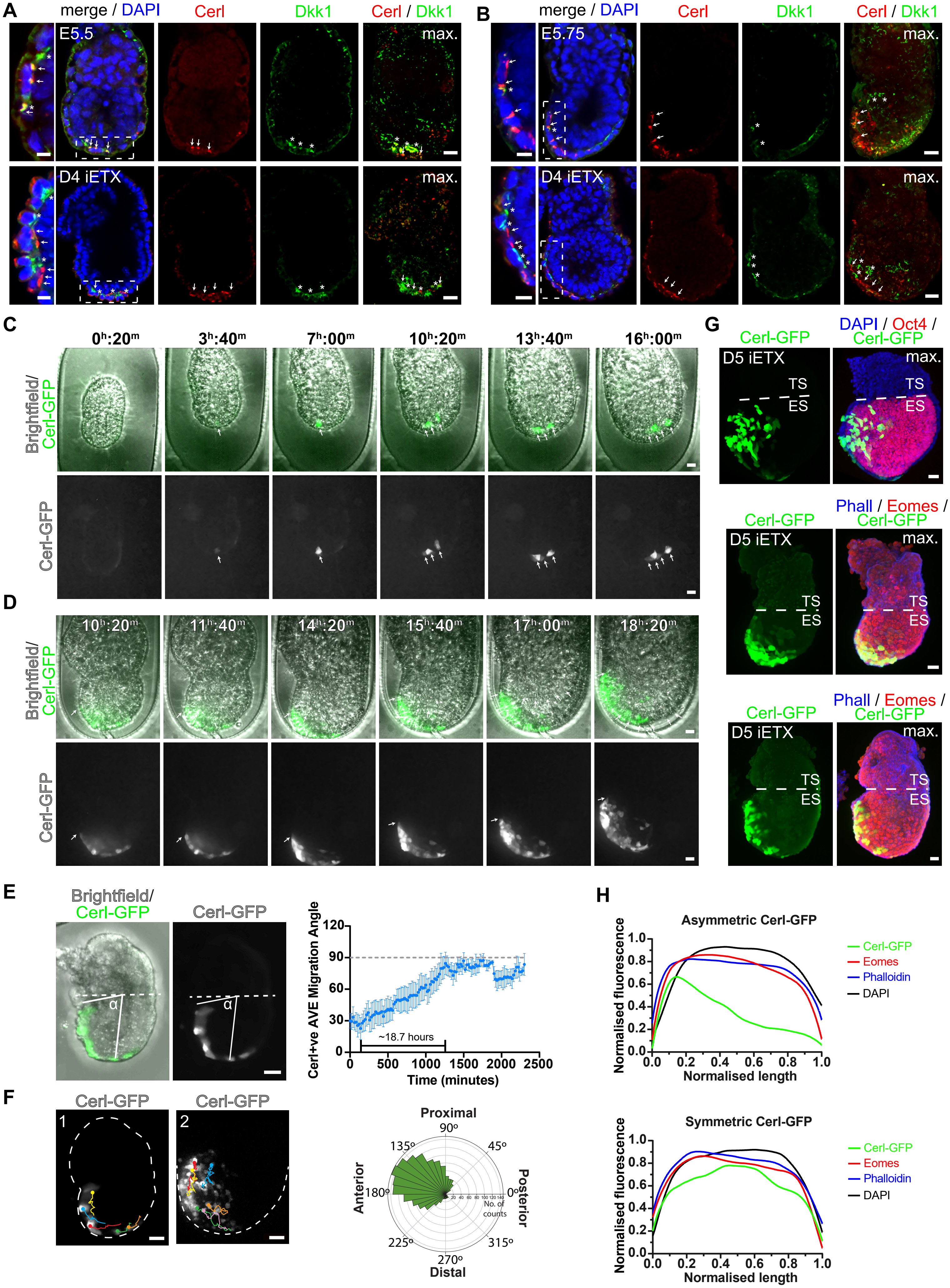

Figure S3. iETX embryos develop a migrating anterior visceral endoderm, related to Figure 3. A. (Top) E5.5 wildtype embryo and (bottom) representative iETX embryo at day 4 analysed for Cerl (red, arrows), Dkk1 (green, asterisks), which are localized at the distal tip, and DAPI (blue). Embryo: 2 examples. iETX embryo: 19/25 from 5 independent experiments. Scale bar, 30 µm. Scale bar of side panels, 10 µm. **B.** (Top) E5.75 wildtype embryo and (bottom) representative iETX embryo at day 4 analysed for Cerl (red, arrows), Dkk1 (green, asterisks), which are localized on one side, and DAPI (blue). Embryo: 3 examples. iETX embryo: 6/25 from 5 independent experiments. Scale bar, 30 µm. Scale bar of side panels, 10 µm. **C.** Time lapse movie of AVE formation in a Cerl-GFP reporter iETX embryo captured from day 3 of development. Top row: Cerl-GFP positive cells are in green, every other cell is in grey. Bottom row: Cerl-GFP-positive cells are in grey. Arrows highlight Cerl-GFP positive cells. For DVE induction, 34 from 3 separate experiments were examined; of these, 12 showed induction at the tip of the iETX embryo; in 8 instances, GFP signal was already present, hence it was not possible to ascertain its origin; in 12 cases there was no GFP upregulation during imaging; in 2 cases, signal was not induced at the tip. Scale bar, 30 µm. **D.** Time lapse movie of AVE migration in a Cerl-GFP reporter iETX embryo captured from day 3 of development. Top row: Cerl-GFP positive cells are in green, every other cell is in grey. Bottom row: Cerl-GFP-positive cells are in grey. Arrow highlights the Cerl-GFP positive migrating leading cell. For AVE migration, 32 iETX embryos from 3 independent experiments were examined; out of these, 11 showed migration, 10 showed no migration and 11 displayed no signal throughout imaging. Scale bar, 30 µm. **E.** (Left) Cerl-GFP iETX embryo shown in Fig.3Q with a schematic for AVE migration angle measurement. The AVE migration angle was considered as the one comprising the distal tip and the Cerl-GFP+ve cell closest to the ES/TS boundary. Scale bar, 30 µm**.** (Right) Plotting AVE migration angle as a function of time. Error bars represent the standard deviation. The grey dashed line at 90° represents the ES/TS boundary. 4 iETX embryos from 3 independent experiments. **F.** (1) Still-frame image of the iETX embryo from (E), showing the migration trajectories of five Cerl-GFP-positive cells over the course of 3-6.5 hours. Dashed lines show the outline of the iETX embryo. Scale bar, 30 µm. (2) Migration trajectories of six Cerl-GFP-positive cells over the course of 10-12.5 hours of the iETX embryo shown in (D). Scale bar, 50µm. (Right). Polar histogram of the migration directionality of Cerl-GFP-positive cells, calculated using migration trajectories as shown in (1,2) (n = 26 cells from 7 iETX embryos, 2 independent experiments). **G.** Examples of iETX embryos analysed at D5 of development with GFP (green), Oct4 or Eomes (red) and Phalloidin or DAPI (blue); scale bar, 30 µm. Max=maximum projection. The ES and TS compartments and their boundary are indicated. The top iETX embryo is the one shown in E, processed by immunofluorescence after live imaging. **H.** The fluorescent signal of Cerl-GFP (green), Eomes (red), Phalloidin (blue) and DAPI (black) was plotted as a function of length of the ES compartment in iETX embryos at day 5 of development. Cerl-GFP expression was classified as asymmetric if it displayed a single peak on one side of the ES compartment, otherwise it was classified as symmetric (also see Methods). Expression of Eomes, Phalloidin and DAPI was symmetric across the ES compartment in all cases examined. Asymmetric Cerl-GFP expression: 26 iETX embryos / 36, n=3; symmetric Cerl-GFP expression: 10/36, n=3. Scale bar, 30 µm.

Figure S4 (related to Figure 4)

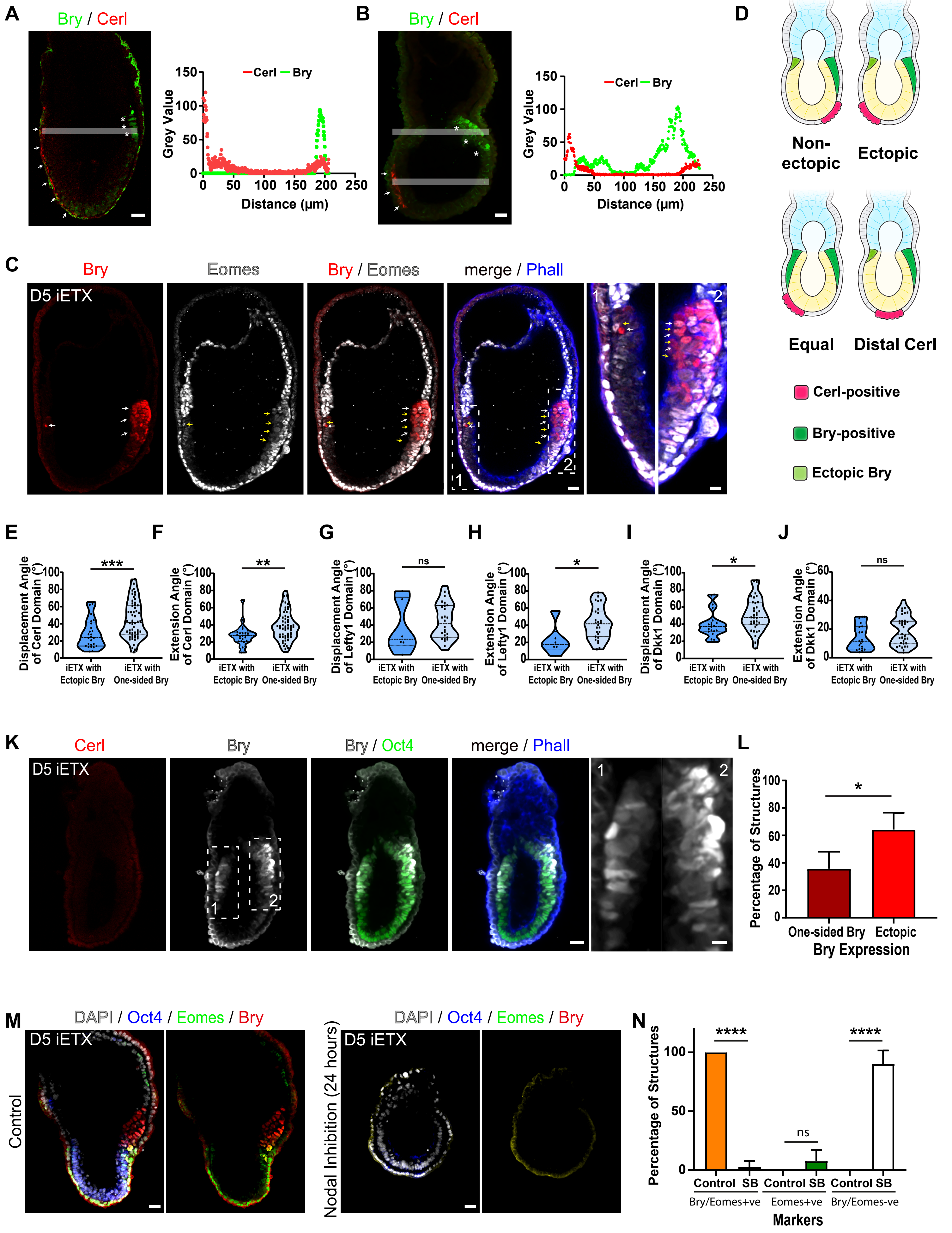

Figure S4. Anterior and posterior domains form on opposite sides of iETX embryos, related to Figure 4. A. Cerl (red) and Bry (green) fluorescent intensity plots as a function of distance along the grey line of the natural embryo, which is the same one as in Fig. 4A. Scale bar, 30 µm. **B.** Cerl (red) and Bry (green) fluorescent intensity plots as a function of distance along the grey lines of the iETX embryo, which is the same one as in Fig. 4B. Scale bar, 30 µm. **C.** Representative iETX embryo at day 5 of development analysed for Bry (red, white arrows), Eomes (grey, yellow arrows) and Phalloidin (blue). 26 / 26, n=3. Scale bar, 30 µm. Dashed rectangles are magnified on the right. Scale bar, 10 µm. **D.** Schematic of possible Bry/Cerl position combinations when, in addition to a proper posterior domain, the iETX embryo shows ectopic expression of posterior markers around the ES/TS boundary. Non-ectopic: Cerl is on the side of the proper posterior domain. Ectopic: Cerl is on the side of the ectopic domain. Equal: both posterior domains are equal. Distal Cerl: Cerl is at the distal tip. **E,F.** Violin plots showing the angle of displacement away from the distal tip and towards the ES-TS boundary of Cerl-positive cells (E) or the angle of extension of the Cerl domain **(**F) when the iETX embryo shows Bry expression on one side only (iETX with one-sided Bry, right violin) or it shows ectopic Bry expression (iETX with ectopic Bry, left violin). Each dot represents an iETX embryo; (E) $p = 0.0010$, Mann-Whitney non-parametric test. (F) $p = 0.0016$, Mann-Whitney non-parametric test. Each graph has 26 samples in the left violin, 57 samples in the right, n=3. **G,H.** Same as the above but with Lefty1 displacement (G) or Lefty1 domain extension (H); (G) p = ns, Mann-Whitney non-parametric test. (H) $p = 0.0252$, Mann-Whitney non-parametric test. Each graph has 6 samples in the left violin, 16 samples in the right, n=3. **I,J.** Same as the above but with Dkk1 displacement (I) or Dkk1 domain extension (J); (I) $p = 0.0215$, Mann-Whitney non-parametric test. (J) p = ns, Mann-Whitney non-parametric test. Each graph has 20 samples in the left violin, 35 samples in the right, n=3. In all violin plots, median and quartiles are graphed**. K.** Representative iETX embryo at day 5 of development analysed for Cerl (red), Bry (grey), Oct4 (green) and Phalloidin (blue). Cerl expression is absent and Bry expression is observed on both sides of the ES compartment. 29 / 46, n=4. Scale bar, 30 µm. Dashed rectangles are magnified on the right, scale bar, 10 µm. **L.** Scoring Bry expression at day 5 of development in iETX embryos that do not express Cerl. If Bry is on one side of the ES compartment, it is scored as “One-sided Bry”; if Bry is on both sides of the ES compartment, it is scored as “Ectopic”. Error bars=SD. The numbers of iETX embryos in each category are: One-sided Bry = 17, Ectopic = 29, n=4. Unpaired Student T-test, $p = 0.0172$. **M.** iETX embryos at 4 days of development were treated with a Nodal inhibitor for 24 hours and analysed at day 5 for Oct4 (blue), Eomes (green), Bry (red) and DAPI (grey). Scale bar, 30 µm. Control: 22 iETX embryos, Nodal inhibition: 35 iETX embryos, n=4 each. **N.** Quantification of the percentage of iETX embryos expressing Bry and Eomes in control conditions or after Nodal inhibition. Structures that were Bry/Eomes+ve, Eomes+ve, or Bry/Eomes-ve were included. There is no condition scoring iETX embryos positive for Bry but negative for Eomes because they were not observed, neither in control, nor in Nodal inhibition treatment. Error bars=SD. One-Way ANOVA and Tukey’s multiple comparison test. Control: 22 iETX embryo, Nodal inhibition: 35 iETX embryos, n=4 each. $p < 0.0001$.

Figure S5 (related to Figure 5)

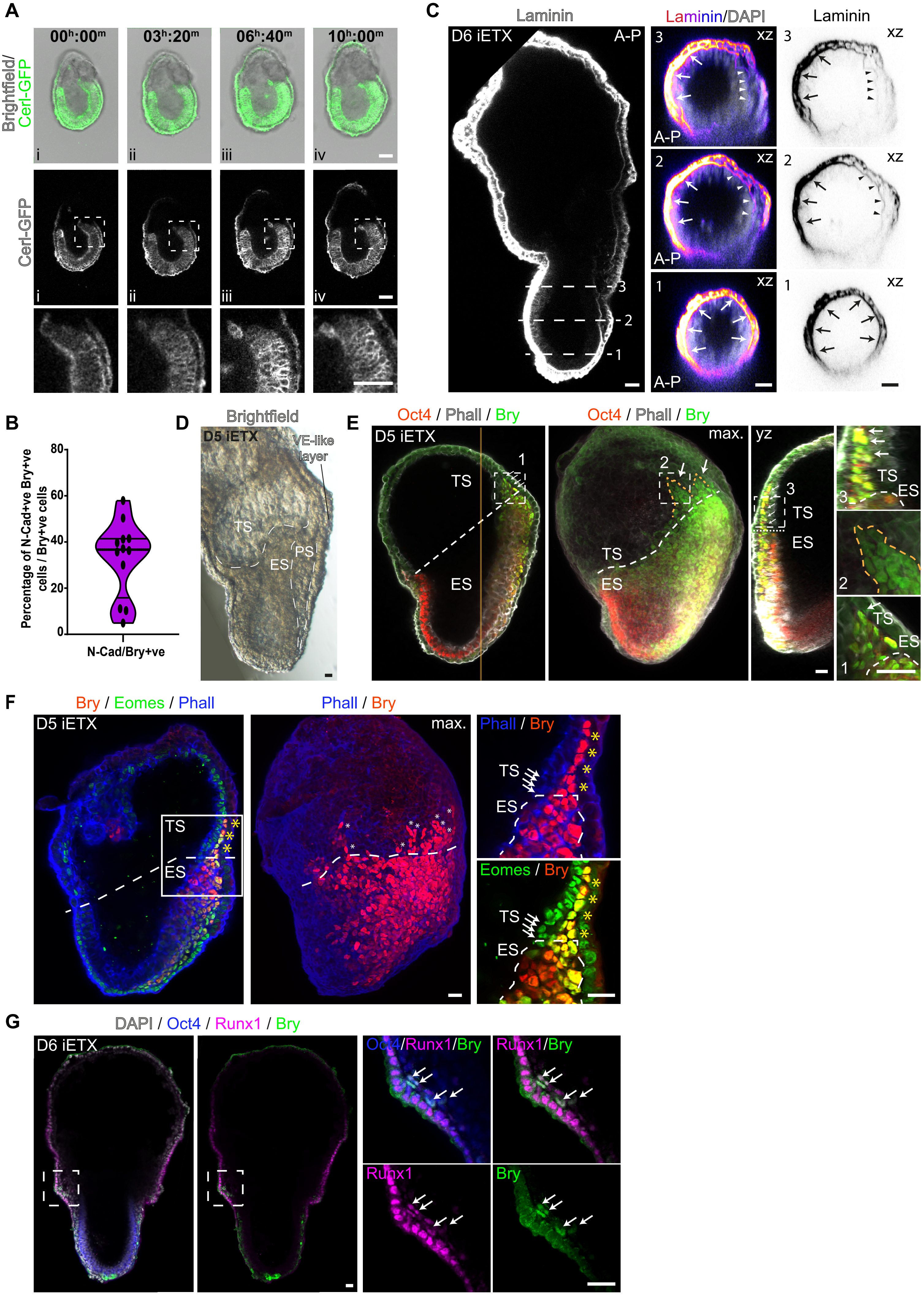

Figure S5. iETX embryos gastrulate and form extra-embryonic mesoderm, related to Figure 5. A. Time-lapse stills of an iETX embryo imaged from day 4 to day 5. Top row: TS cells are in grey, wild-type CAG-GFP and induced CAG-tetOG4 are in green. (Centre row) CAG-GFP cells are displayed in grey. Dashed squares are magnified at the bottom and highlight the prospective posterior side. 6/13 structures with comparable EMT, n=3. Scale bar, 50 µm. **B.** (right) Quantification of the percentage of Bry+ve cells expressing N-Cad in iETX embryos. Each dot represents the percentage in a single iETX embryo. On the violin plot, median and quartiles are shown.12 iETX embryos, n=3. **C.** iETX embryo from Fig. 5G analysed for Laminin; orthogonal sections along the indicated dashed lines highlight laminin breakdown in the posterior. The anterior-posterior axis is indicated. The orthogonal sections display either DAPI (grey) and Laminin (fire) or Laminin (inverted). Arrows indicate intact laminin, arrowheads indicate where laminin is breaking down in the posterior side. 21/24 examples, n=4. Scale bar, 30 µm. **D.** iETX embryo at 5 days of development as observed under a stereo microscope. The TS compartment, ES compartment, primitive streak (PS) and VE-like layer are indicated. The dashed lines highlight the PS and the ES/TS boundary. Scale bar, 30 µm. 76/139, n=6. **E.** iETX embryo at 5 days of development analysed for Bry (green), Oct4 (red) and Phalloidin (Grey). (Left) The dashed lines indicate the ES/TS boundary; arrows indicate Bry-positive in the TS compartment and that area is magnified in (1). (Centre) Maximum projection; ES/TS boundary is indicated and patches of Bry-positive cells in the TS compartment are indicated by orange dashed lines and arrows and magnified in (2). (Right) YZ orthogonal panel generated along the orange line; arrows highlight Bry-positive cells in the TS compartment magnified in (3). 20/44 structures from 4 independent experiments. Scale bar, 30 µm. **F.** iETX embryo at 5 days of development analysed for Bry (red), Eomes (green) and Phalloidin (blue). (Left) The dotted lines indicate the ES/TS boundary. The area in the square is magnified on the right; the dashed line and the arrows indicate the ES/TS boundary, asterisks indicate Bry-positive cells of ES origin in the TS compartment. (Centre) Asterisks indicate Bry-positive cells in the TS compartment. Max.=maximum projection. 20/44 from 4 independent experiments. Scale bar, 30 µm. **G.** iETX embryo at 6 days of development analysed for Runx1 (magenta), Bry (green), Oct4 (blue) and DAPI (grey). The dashed square is magnified on the right. Arrows highlight co-expression of Runx1 and Bry. Scale bar, 30 µm. 4/17, n=3.

Figure S6 (related to Figure 6)

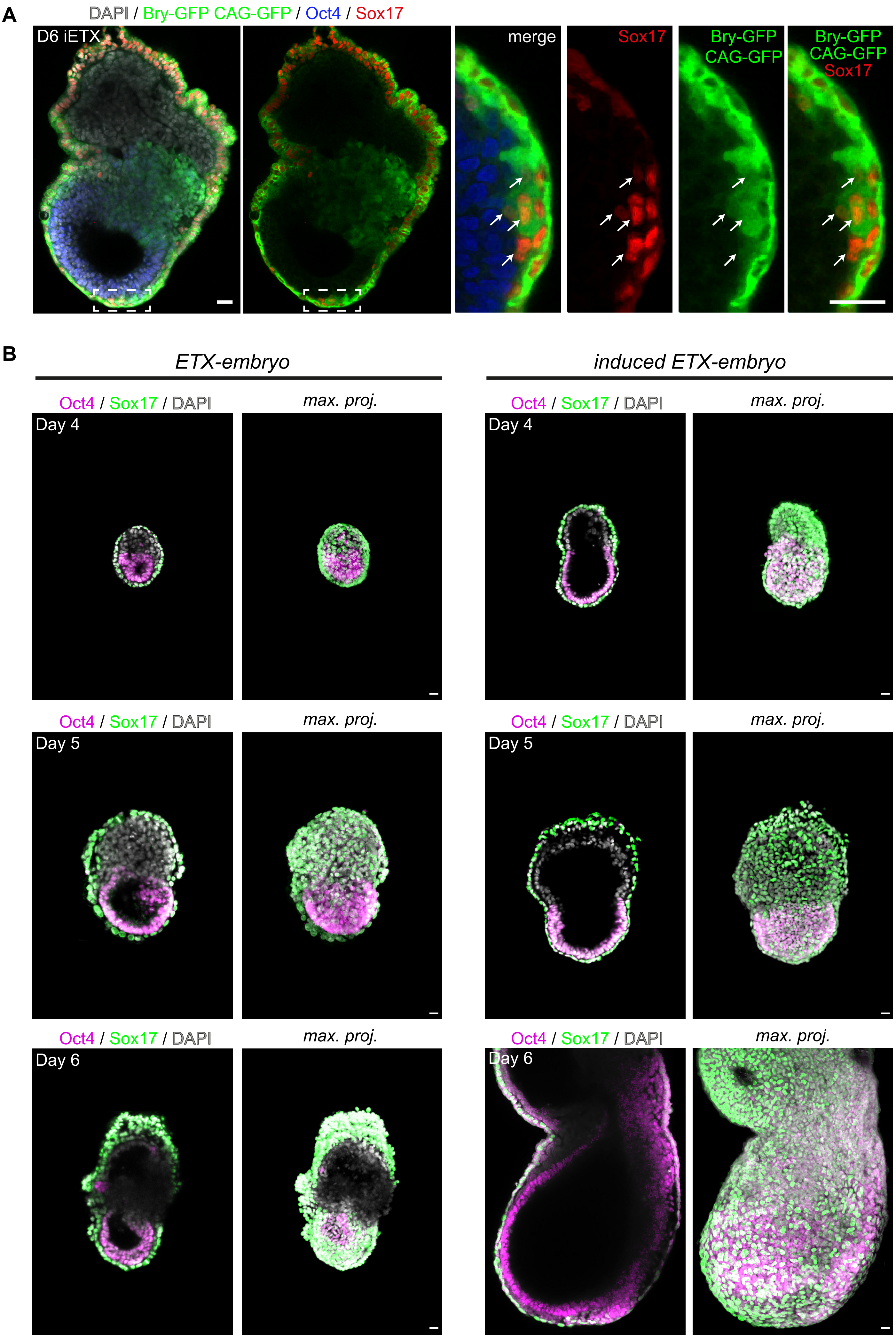

Figure S6. Comparison of ETX embryos and iETX embryos, related to Figure 6. A. iETX embryo at 6 days of development and generated with a Bry-GFP ES line was analysed for Oct4 (blue), Sox17 (red), Bry-GFP and CAG-GFP (green, αGFP antibody) and DAPI (grey). Dashed area is magnified on the right. Arrows highlight cells co-expressing Sox17 and Bry-GFP. Scale bar, 30 μm. 6/7, n=2. **B.** Comparison of ETX embryos and iETX embryo side by side at 4, 5 and 6 days of development, analysed for Oct4 (magenta), Sox17 (green) and DAPI (grey); max. proj.=maximum projection. In the panel of ETX embryo at day 6 and iETX embryo at day 5, part of another structure was visible in the original image and was cropped out. Scale bar, 30 μm.

Figure S7 (related to Figure 7)

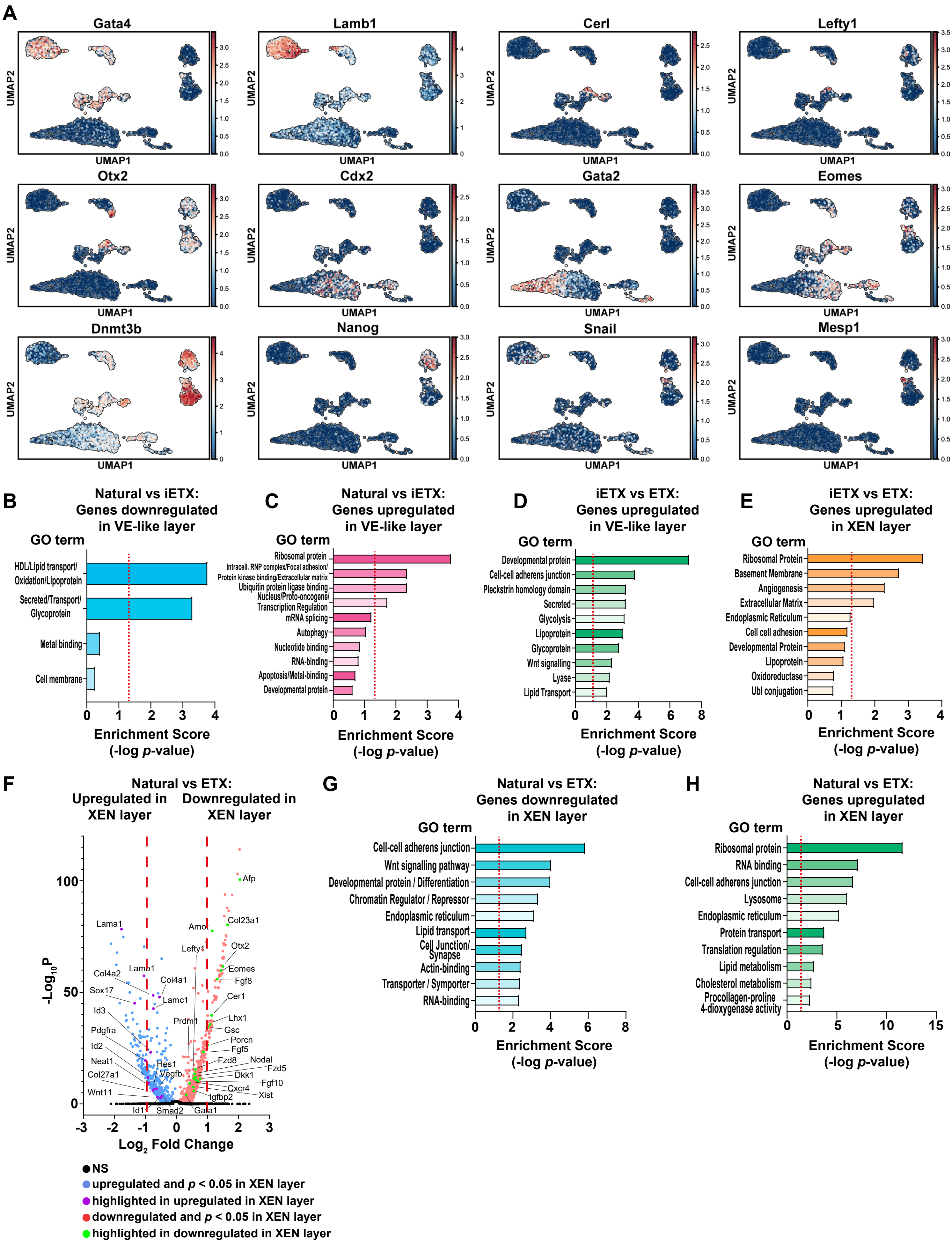

Figure S7. Single cell sequencing shows similarity between the VE-like layer of iETX embryos and the natural visceral endoderm, related to Figure 7. A. Panel of single cell sequencing data UMAP for the selected genes utilised to identify the subpopulations shown in Figure 7D, in combination with leiden clusters. Relative gene expression levels are shown in shades of blue and red. **B.** Gene Ontology analysis of genes downregulated in the VE-like layer of iETX embryos when compared to the visceral endoderm of natural embryos. **C.** Gene Ontology analysis of genes upregulated in the VE-like layer of iETX embryos in comparison to the visceral endoderm of natural embryos. **D.** Gene Ontology analysis of genes upregulated in the VE-layer of iETX embryos in comparison to the XEN layer of ETX embryos. **E.** Gene Ontology analysis of genes upregulated in the XEN layer of ETX embryos in comparison to the VE-like layer of iETX embryos. **F.** Volcano plot of genes downregulated and upregulated in the XEN layer of ETX embryos in comparison to the visceral endoderm of natural embryos. **G.** Gene Ontology analysis of genes downregulated in the XEN layer of ETX embryos when compared to the visceral endoderm of natural embryos. **H.** Gene Ontology analysis of genes upregulated in the XEN layer of ETX embryos in comparison to the visceral endoderm of natural embryos.

Table S1. Comparison of the developmental milestones of iETX embryos and ETX embryos, related to Figure 1-6 and Figure S1-S6.

Developmental Landmarks	induced ETX embryo	ETX embryo (Sozen et al., 2018)
Cylindrical morphology and proximal-distal elongation	22% (960/4410)*	20% (87/432)*
Eomes expression in embryonic VE-like compartment	97% (41/42)	20% (2/10)
Otx2 expression in embryonic VE-like compartment	87% (27/31)	42% (12/28)
Anterior visceral endoderm formation	70%** (52/73)	41%** (7/17)
Anterior visceral endoderm migration	33% (11/32)	N/A ##
Cerl/Bry expression	56% (68/122)	N/A**
Bry expressing domain formation (regionalised mesoderm)	55% (38/69)	42% (42/101)
Epithelial-Mesenchymal-Transition (EMT)	40-60% (27/42)	40% (20/50)
Collective EMT and primitive streak elongation for full gastrulation	40-60% (27/42)	N/A ***
Extra-embryonic mesoderm formation	45% (20/44)	N/A ***
Runx1 expression in extra-embryonic mesoderm	23% (4/17)	N/A ***
Primitive streak heterogeneity - Cerl	51% (17/33)	N/A ***
Primitive streak heterogeneity - Lefty1	79% (27/34)	N/A ***
Primitive streak heterogeneity - Dkk1	37% (21/56)	N/A ***
Axial mesoderm formation	71% (37/52)	20% (8/42)
Definitive endoderm formation	55% (17/31)	40% (16/42)

Notes

* The denominators in this row represent all the structures examined and the numerators represent the structures that passed the criteria for inclusion. In the rows below, the denominators represent the number of structures that passed the criteria for inclusion and the numerators represent the number of structures with the hallmark/trait being analysed

**Only Lefty1 in AVE of ETX embryos; in iETX, 70% at D5 with Dkk1 and Cerl (133/171), Lefty1 drops to 40% (32/87)

ETX with Cerl-GFP XEN cells could not be generated

*** In ETX embryos streak formation does not progress past initial EMT, hence more advanced processes, with the exception of definitive endoderm formation, could not be observed

Table S2. Genes involved in Wnt signalling identified by Gene Ontology analysis as upregulated in the VE-like layer of iETX embryos in comparison to the XEN layer of ETX embryos. Gene symbol, protein name and Wnt-related annotation on the Uniprot database are reported, related to Figure 7 and Figure S7.

Gene Symbol	Protein Name	Wnt-related Annotation (Uniprot)
Lgr5	Leucine-rich repeat-containing G-protein coupled receptor 5	Wnt upregulation
Sfrp5	Secreted frizzled-related protein 5	Negative Wnt regulation
Cthrc1	Collagen triple helix repeat-containing protein 1	Negative regulation of collagen deposition and Wnt signalling
Fzd8	Frizzled-8	Wnt receptor
Sfrp1	Secreted Frizzled-related protein 1	Negative Wnt regulation
Trabd2b	Metalloprotease TIKI2	Negative Wnt regulation
Axin2	Axin-2	Inhibitor of Wnt signalling, β-Catenin downregulation
Fzd5	Frizzled-5	Wnt receptor
Porcn	Protein-serine O-palmitoleoyltransferase Porcupine	Wnt signalling regulator
Hhex	Hematopoietically-expressed homeobox protein Hhex	AVE marker; initial Wnt enhancement and successive Nodal inhibition
Dkk1	Dickkopf-related protein 1	AVE marker; Wnt inhibitor
Dact1	Dapper homolog 1	Involved in both positive and negative regulation of Wnt
Rspo3	R-spondin-3	Binder of Lgr5
Fermt2	Fermitin family homolog 2	Regulator of transcription in Wnt signalling
Gpc4	Glypican-4	Wnt signalling
Prickle2	Prickle-like protein 2	Wnt signalling
Bambi	BMP and Activin membrane-bound inhibitor homolog	Negative regulation of TGF-β signalling, positive regulation of Wnt
Fgf8	Fibroblast growth factor 8	Cooperation with Wnt1
Sox4	Transcription factor Sox-4	Positive Wnt regulation
